# Artificial Intelligence in cancer epigenomics: a review on advances in pan-cancer detection and precision medicine

**DOI:** 10.1186/s13072-025-00595-5

**Published:** 2025-06-14

**Authors:** Karishma Sahoo, Prakash Lingasamy, Masuma Khatun, Sajitha Lulu Sudhakaran, Andres Salumets, Vino Sundararajan, Vijayachitra Modhukur

**Affiliations:** 1 Integrative Multiomics Lab, School of Bio Sciences and Technology, Vellore Institute of Technology, Vellore, 632014 Tamil Nadu India; 2 Department of Obstetrics and Gynecology, Institute of Clinical Medicine, University of Tartu, L. Puusepa 8, 50406 Tartu, Estonia; 3 Celvia CC AS, 50411 Tartu, Estonia; 4 Department of Obstetrics and Gynecology, University of Helsinki and Helsinki University Hospital, Haartmaninkatu 8, 00290 Helsinki, Finland; 5 Division of Obstetrics and Gynecology, Department of Clinical Science, Intervention and Technology (CLINTEC), Karolinska Institute, and Karolinska University Hospital, 14183 Huddinge, Sweden

**Keywords:** DNA methylation, Artificial intelligence, Machine learning, Deep learning, Cancer epigenomics, Pan-cancer, Early detection, Precision oncology, Liquid biopsy, Multi-omics, Multi-cancer diagnostics

## Abstract

DNA methylation is a fundamental epigenetic modification that regulates gene expression and maintains genomic stability. Consequently, DNA methylation remains a key biomarker in cancer research, playing a vital role in diagnosis, prognosis, and tailored treatment strategies. Aberrant methylation patterns enable early cancer detection and therapeutic stratification; however, their complex patterns necessitates advanced analytical tools. Recent advances in artificial intelligence (AI) and machine learning (ML), including deep learning networks and graph-based models, have revolutionized cancer epigenomics by enabling rapid, high-resolution analysis of DNA methylation profiles. Moreover, these technologies are accelerating the development of Multi-Cancer Early Detection (MCED) tests, such as GRAIL’s Galleri and CancerSEEK, which improve diagnostic accuracy across diverse cancer types. In this review, we explore the synergy between AI and DNA methylation profiling to advance precision oncology. We first examine the role of DNA methylation as a biomarker in cancer, followed by an overview of DNA profiling technologies. We then assess how AI-driven approaches transform clinical practice by enabling early detection and accurate classification. Despite their promise, challenges remain, including limited sensitivity for early-stage cancers, the black-box nature of many AI algorithms, and the need for validation across diverse populations to ensure equitable implementation. Future directions include integrating multi-omics data, developing explainable AI frameworks, and addressing ethical concerns, such as data privacy and algorithmic bias. By overcoming these gaps, AI-powered epigenetic diagnostics can enable earlier detection, more effective treatments, and improved patient outcomes, globally. In summary, this review synthesizes current advancements in the field and envisions a future where AI and epigenomics converge to redefine cancer diagnostics and therapy.

## Introduction

Cancer remains the second leading cause of mortality worldwide and is responsible for nearly 10 million deaths annually [1]. Despite substantial advancements in oncology, early detection and personalized treatment continue to pose major challenges. Traditional diagnostic methods, including histopathology, imaging, and tissue biopsies, often detect cancer only at advanced stages, limiting therapeutic options and reducing survival rates [2]. Moreover, the inherent heterogeneity of cancer within and between patients further complicates the development of universal diagnostic and therapeutic strategies [3]. Epigenetic modifications, particularly DNA methylation, have emerged as stable and highly sensitive tumor-type-specific biomarkers with potential applications across all stages of clinical disease management, including risk assessment, early diagnosis, treatment management, and post-treatment monitoring. These biomarkers play a crucial role in prognosis prediction and therapy monitoring, making them valuable tools for precision medicine [4, 5]. DNA methylation involves the addition of a methyl group to cytosine residues (5-methylcytosine, 5mC) at CpG dinucleotides, serving as a fundamental epigenetic mechanism that controls gene expression and maintains genomic stability [6]. In healthy cells, DNA methylation patterns are tightly regulated by DNA methyltransferases (DNMTs), which add methyl groups, and ten-eleven translocation (TET) enzymes, which remove them. These patterns are essential for normal cellular functions, including differentiation, development, and X-chromosome inactivation. However, in cancer, global hypomethylation and locus-specific hypermethylation disrupt these gene regulatory mechanisms, leading to the silencing of tumor suppressor genes (e.g., *VHL*, *p16*) and the activation of oncogenes (e.g., *MYC*, *RAS*) [7]. These aberrant methylation patterns are not only hallmarks of tumorigenesis but also stable and detectable in circulating tumor DNA (ctDNA), making them ideal biomarkers for non-invasive, liquid biopsy-based cancer diagnostics [8, 9].

The advent of high-throughput sequencing technologies has revolutionized DNA methylation profiling, enabling single-base resolution across the genome. Techniques such as whole-genome bisulfite sequencing (WGBS) and methylation arrays (e.g., Illumina Infinium) have generated vast datasets, revealing methylation signatures linked to specific cancer types, stages, and therapeutic responses [10]. However, the sheer volume and complexity of these datasets pose significant challenges for conventional analytical methods. To address this, artificial intelligence (AI) and machine learning (ML) have emerged as transformative powerful tools for analyzing the epigenetic landscape of tumors with unprecedented precision and efficiency. Advanced cutting-edge AI algorithms, including convolutional neural networks (CNNs) and gradient boosting machines (GBMs), enhance the ability to recognize cancer-specific methylation patterns, paving the way for pan-cancer screening and tumor tissue-of-origin (TOO) prediction [11, 12].

AI-powered methylation analysis has led to the development of multi-cancer early detection (MCED) tests, which analyze circulating tumor DNA (ctDNA) methylation patterns to detect multiple cancer types from a single blood test. Notable advancements include, GRAIL’s Galleri test employs targeted methylation sequencing and ML algorithms to detect over 50 types of cancer and their TOO with high specificity and accuracy [13]. Similarly, CancerSEEK integrates gene mutational data and protein biomarkers to improve diagnostic sensitivity across eight cancer type [14]. These groundbreaking innovations represent a paradigm shift in cancer diagnostics, offering earlier detection, improved patient outcomes, and reduced healthcare costs. Despite these advancements, several challenges hinder widespread clinical adoption. The interpretability of AI models, often called the "black-box" problem, limits their clinical adoption [15]. It is also important to address ethical issues like data privacy and algorithmic bias to ensure fair and equitable access to these technologies.

Furthermore, population-specific methylation variations and dynamic nature of the tumor epigenome complicate the development of universal biomarkers. Future research must prioritize explainable AI (XAI), integrate multi-omics data (genomics, transcriptomics, proteomics), and validate findings across large, multi-ethnic cohorts to enhance accuracy, equity, and clinical implementation.

**Fig. 1:**
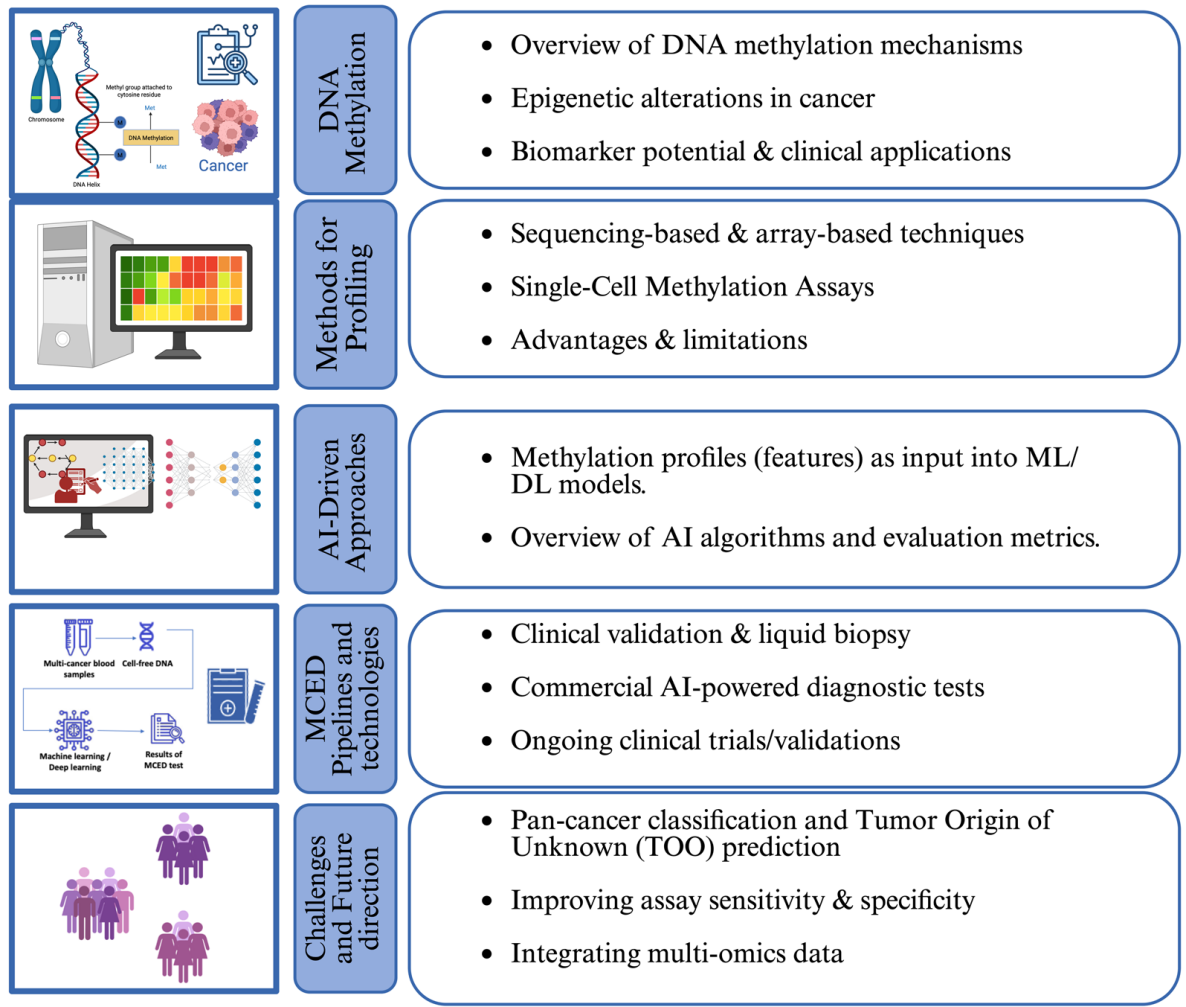
Schematic flow diagram of the review design. The schema depicts five main phases of the review process, including DNA methylation and its significance, Profiling methods, AI-driven cancer detection, MCED pipelines and technologies, and Challenges and future directions

This review is organized as follows: Section "DNA methylation in cancer: mechanisms, biomarker potential, and clinical applications" provides an overview of DNA methylation mechanisms and their role in cancer, discussing epigenetic alterations, biomarker potential, and clinical applications. Section "Methods for DNA methylation profiling:" outlines methodologies for DNA methylation profiling, covering sequencing-based and array-based techniques, along with their advantages and limitations. Section "AI Techniques for cancer prediction using DNA methylation" explores AI-driven approaches for methylation-based cancer detection, including machine learning (ML) and deep learning (DL) models tailored for pan-cancer classification and TOO prediction using DNA methylation. Section "Multi-cancer early detection (MCED): pipelines, technologies and industry advancement" examines MCED pipelines, technologies, and industry advancements, focusing on clinical validation efforts, emerging liquid biopsy frameworks, and commercial AI-powered diagnostic tests. Section "Future directions, limitation and concluding remarks" discusses key challenges and future directions, including strategies for improving sensitivity, integrating multi-omics data, and addressing ethical and regulatory considerations. It also provides concluding remarks on the impact of AI-driven DNA methylation analysis in advancing precision oncology and outlines future research priorities for clinical translation. Figure 1 gives a schematic representation of the review design.

## DNA methylation in cancer: mechanisms, biomarker potential, and clinical applications

DNA Methylation as a Diagnostic and Prognostic Biomarker: To shed light on the potential of AI-driven methylation diagnostics, it is crucial to understand the fundamental mechanisms of DNA methylation and how these epigenetic modifications contribute to cancer progression. A prognostic biomarker indicates the likely progression of a patient’s cancer, independent of treatment. On the other hand, a predictive biomarker provides insight into the potential effectiveness of a specific therapy and may also serve as a therapeutic target [17]. Strikingly, DNA methylation patterns alone can serve as both prognostic and diagnostic biomarker for several diseases, including cancer within an individual’s genome. These biomarkers offer several advantages in disease diagnosis due to their stability, cost-effective amplification, and specificity to localized regions of DNA methylation [16]. Moreover, FDA-approved diagnostic tests utilizing methylation biomarkers have demonstrated high sensitivity and specificity, enabling non-invasive detection of early-stage cancers. For instance, *SEPT9* methylation serves as a biomarker for colorectal cancer, while *BMP3/NDRG4* methylation has shown high efficacy in pancreatic cancer detection [18–20]. Additionally, several methylation markers are undergoing clinical evaluation, including *SHOX2* for lung cancer and *RASSF1A, RARB2*, and *GSTP1* for lung, breast, genitourinary, and colorectal cancers [21].

**Fig. 2:**
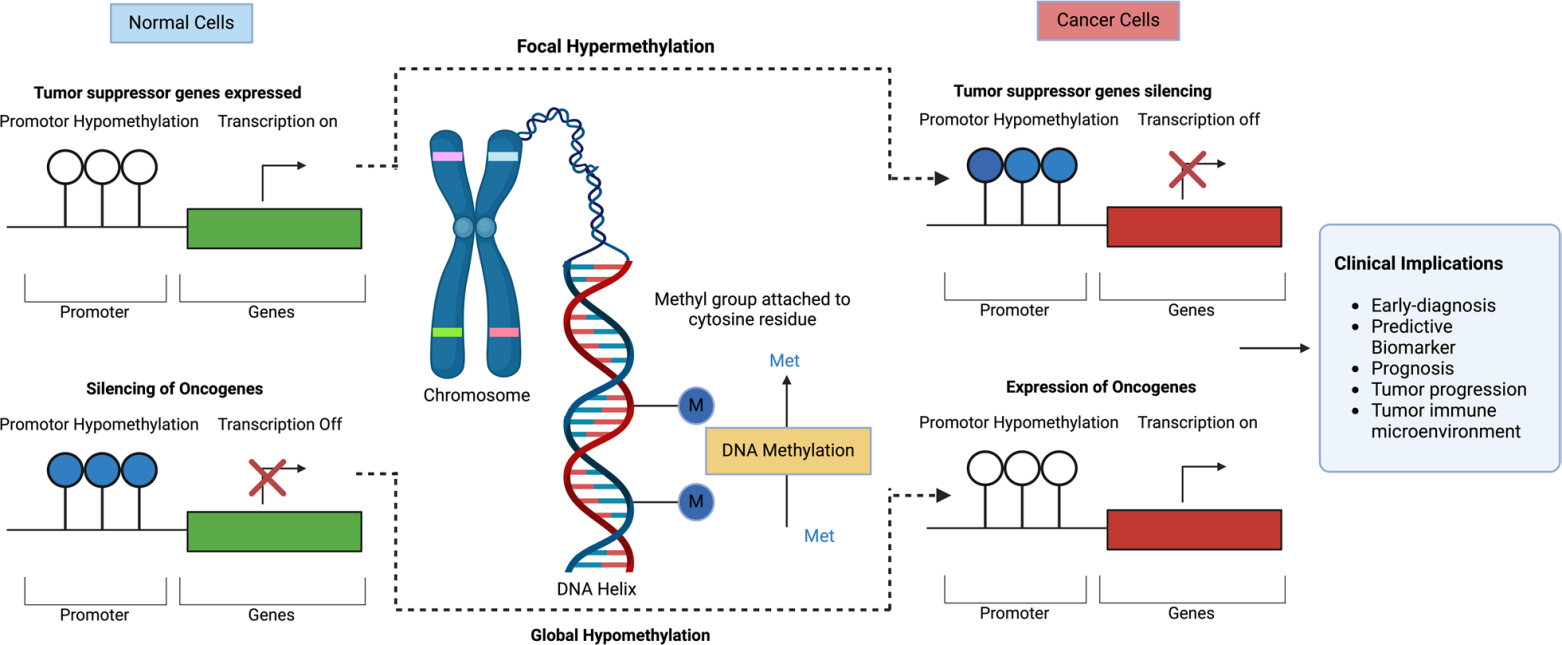
Mechanism of aberrant DNA methylation and its impact on Cancer Cell proliferation: In healthy cells, promoter hypomethylation activates tumor suppressor genes, while hypermethylation inactivates oncogenes. Conversely, in cancerous cells, hypermethylation silences tumor-suppressing genes, and hypomethylation activates cancer-promoting genes. These epigenetic alterations contribute to cancer-related processes and can be accessed for early detection, prognosis, biomarker identification, understanding tumor microenvironment dynamics, and assessing disease progression

DNA Methylation and Tumorigenesis: Aberrant DNA methylation contributes to tumorigenesis by disrupting gene expression and genomic stability. Hypermethylation in CpG islands of tumor suppressor genes (TSGs) leads to gene silencing, while global hypomethylation activates oncogenes and promotes chromosomal instability. These alternations often involve the functioning of DNMT and demethylase (MBD2), whose elevated expression can induce hypermethylation for silencing TSGs in CpG islands [22]. In the early-stage neoplasia, global hypomethylation in intergenic and intronic regions can occur passively through DNMT1 loss or actively via the oxidation of methylcytosine mediated by TET enzymes, followed by base excision repair [23]. The consequent genomic instability and chromosomal abnormalities promote carcinogenesis and contribute to immune infiltration [24]. Moreover, promoter region hypermethylation often silences tumor-suppressor genes, leading to tumor progression, treatment resistance, and reduced survival rates [25]. Figure 2 illustrates the key mechanisms of aberrant DNA methylation dynamics in normal and cancer cells, emphasizing its role in tumor proliferation and clinical implications.

In addition to early diagnosis, aberrant DNA methylation patterns also serve as biomarkers for disease staging, prognosis, and therapy response monitoring [16]. For instance, Gu X *et al*. 2022 developed a prognostic model using distinct methylated gene profiles in circulating tumor cells of lung adenocarcinoma, uncovering notable disparities in biological processes, tumor microenvironment, genetic alterations, and clinical outcomes [26]. DNA methylation patterns offer valuable insights into patient responses to specific treatments and can serve as predictive biomarkers, providing guidance on expected efficacy of therapeutic interventions. In this regard, Lee *et al*. 2022 reported that *DNMT1* overexpression correlates with radioresistance in head and neck squamous cell carcinoma (HNSCC), suggesting its potential as a biomarker for predicting the effectiveness of CD47 antibody-based therapy in recurrent HNSCC following radiotherapy [27].

DNA Methylation in Metastasis and Epithelial-to-Mesenchymal Transition (EMT): In exploring the role of DNA methylation in tumor progression, which predominantly results in transcriptional silencing, several studies have suggested DNA hypermethylation-induced silencing of TSGs such as *VHL* and metastasis-suppressing genes such as *E-cadherin* in lung and metastatic breast cancer, respectively [28, 29]. Recent studies have linked elevated DNA methylation levels in genes associated with EMT to an increased likelihood of metastasis. For example, Luo *et al*. 2022 identified significant differences in the promoter methylation patterns, including hypermethylation of *RASGRF2*, *AKR1B1*, *CRMP1*, and hypomethylation of *RHOF* genes in breast cancer tissues with positive lymph nodes compared to those with negative lymph nodes [30]. Additionally, aberrant methylation patterns in the *AKR1B1, RASGRF2, CRMP1, BNIP3, GSTP1, HOXA5*, and *PAX6* genes have been observed in estrogen receptor (ER)-positive and *HER2*-negative breast cancer with axillary lymph node metastasis (ALNM), suggesting their potential as therapeutic targets [30]. Similarly, analysis of sequencing data from hepatocellular carcinoma (HCC) patients suggested the pivotal role of gene body hypermethylation-activated *EMX1-FL* (the full-length protein isoform of *EMX1*) in promoting tumorigenesis and metastasis through EGFR-ERK signaling pathway [31]. Table 1 summarizes the key DNA methylation alterations associated with tumor progression and metastasis across various cancer types. It highlights specific genes, their methylation status (hypermethylation or hypomethylation), and their functional roles in tumor development.

**Table 1:** DNA Methylation Alterations in Tumor Progression and Metastasis: Table summarizes key genes exhibiting altered methylation patterns, their roles in tumor progression, and their clinical significance across cancer types

Gene	Methylation status	Cancer type	Role in tumor progression	References
*VHL*	Promoter Hypermethylation	Lung Cancer	Tumor suppressor silencing, promotes tumorigenesis.	[28]
E-cadherin (*CDH1*)	Promoter Hypermethylation	Metastatic Breast Cancer	Loss of cell adhesion, enhances metastasis	[28]
*RASGRF2*	Promoter Hypermethylation	Breast Cancer (Lymph Node+)	Associated with EMT and metastasis	[30]
*AKR1B1*	Promoter Hypermethylation	Breast Cancer (Lymph Node+, ER+/HER2−)	Linked to ALNM and EMT progression	[30]
*CRMP1*	Promoter Hypermethylation	Breast Cancer (Lymph Node+)	EMT-associated, metastasis regulator	[30]
*RHOF*	Promoter Hypomethylation	Breast Cancer (Lymph Node+)	Potential driver of EMT and metastasis	[30]
*BNIP3*	Promoter Hypermethylation	Breast Cancer (ER+/HER2−, ALNM)	Apoptosis regulation, metastasis-linked	[30]
*GSTP1*	Promote Hypermethylation	Breast Cancer (ER+/HER2−, ALNM)	Detoxification enzyme, methylation linked to tumor progression	[30]
*HOXA5*	Promoter Hypermethylation	Breast Cancer (ER+/HER2−, ALNM)	Transcription factor, EMT-associated	[30]
*PAX6*	Promoter Hypermethylation	Breast Cancer (ER+/HER2−, ALNM)	Regulates cell differentiation, linked to metastasis	[30]
*EMX1-FL*	Gene-body Hypermethylation	Hepatocellular Carcinoma (HCC)	Activates EGFR-ERK signaling pathway, promotes tumorigenesis and metastasis	[31]

** a. EMT: Epithelial-Mesenchymal Transition; **b.** ALNM: Axillary Lymph Node Metastasis; **c.** *EGFR-ERK* signaling pathway: Epidermal Growth Factor Receptor (EGFR) activated Extracellular-signal Regulated Kinase (ERK); **d.** ER+: Estrogen Receptor Positive; **e.** HER2−: Human Epidermal Growth Factor Receptor 2 Negative

DNA Methylation and the Tumor Immune Microenvironment (TIME): Targeting the DNA methylation status within the tumor immune microenvironment (TIME) has emerged as a powerful analytical tool aiming to enhance immune cytotoxicity and reduce immunosuppression by regulating immune cell infiltration, functions, and responses [32]. However, the dynamic remodeling of DNA methylation and subsequent TIME alteration can be considered potential predictors of tumor response to tumor immunotherapy, chemotherapy, and radiotherapy[33]. The analysis of the correlation between the TIMEscore and immune cell infiltrations indicates that patients with high TIMEscore may exhibit increased sensitivity to immunotherapy [34]. In another study by Yu R *et al.* 2023, immune cell infiltration scores, DNA mutation, and copy number variation (CNV) patterns in different subgroups of lung adenocarcinoma (LUAD), based on immune-related methylation sites, provide valuable insights into clinical features, survival outcomes, immune cell infiltration, genomic variations and stem cell characteristics [35].

## Methods for DNA methylation profiling

DNA methylation profiling has significantly enhanced precision in cancer diagnostics and epigenetic research. Genome-wide analysis of DNA methylation patterns, combined with ML techniques, has led to clinical-grade classifiers for early cancer detection [36]. Two primary technologies are used for methylation signals detection: sequencing-based and array-based methods. Prior to the advent of high-throughput sequencing, methylation arrays like Illumina Infinium were the most commonly used method for detecting these signals [37, 38].

### Sequencing-based methods

A range of experimental methods are utilized to analyze DNA methylation in genomic DNA, including whole-genome bisulfite sequencing, pyrosequencing, Nanopore DNA sequencing, methylated DNA immunoprecipitation (MeDIP), Illumina Infinium DNA methylation, targeted bisulfite sequencing with TruSeq Methyl Capture, and ultra-high-performance liquid chromatography combined with mass spectrometry (UHPLC-MS/MS) [39–41].

#### Bisulfite sequencing based methods

Bisulfite Sequencing (BS-Seq) is the gold standard for methylation profiling, as it converts unmethylated cytosines to uracils while leaving methylated cytosines unchanged, enabling single-base resolution detection. WGBS offers comprehensive genome wide coverage (~28 million CpGs) but is limited by high costs, requires high DNA input, limiting its scalability and potential DNA degradation challenges [42]. Another such targeted sequencing method is TruSeq EPIC sequencing providing targeted coverage of 3.34 million CpG sites, outperforming EPIC-array capabilities by demonstrating significant improvement in genomic resolution and coverage [43]. Reduced Representation Bisulfite Sequencing (RRBS): Selectively enriches CpG-dense regions using methylation-insensitive enzymes (e.g., MspI), covering 85% of CpG islands, making it cost-effective but biased toward promoter regions [44].

Affinity Enrichment-Based Methods: Methylated DNA Immunoprecipitation (MeDIP): Enriches methylated DNA using anti-5mC antibodies or anti-methylcytosine binding proteins (MBD), ideal for low-input samples. It covers about 10% of the genome. Notably, RRBS covers 85% of CGIs, especially in promoter regions [45].

Methylation-Sensitive Restriction Enzyme (MSRE) Digestion: Selectively digests unmethylated CpG sites, allowing for comparative methylation analysis, as seen in IMPRESS, a novel multi-cancer detection assay [46]. Some notable limitations of the MSRE method include its ease of use but reduced effectiveness for intermediate methylation levels and relatively high cost. Despite its high specificity, its dependency on specific restriction sites limits its ability to provide comprehensive methylation profiling [47, 48].

Emerging Technologies: Nanopore-Based DNA Sequencing: Directly detects 5mC and 5hmC modifications without bisulfite conversion, reducing DNA degradation issues [49]. Ultra-High Performance Liquid Chromatography–Mass Spectrometry (UHPLC-MS/MS): Provides quantitative methylation analysis at high sensitivity but is unsuitable for genome-wide applications [50].

### Array-based methods

DNA hybridization microarrays offer a cost-effective, rapid analysis, and extensive coverage of predetermined CpG sites. It is widely applied in large-scale population studies such as The Cancer Genome Atlas Consortium (TCGA) [51] and The Genotype-Tissue Expression (GTEx) [52].

#### Illumina infinium beadchip

Array-based method typically uses bisulfite-conversion of DNA to distinguish unmethylated cytosines, appearing as thymines, while 5-methylcytosines remain unchanged, in the amplified sense strand sequence at the single nucleotide level. Originally, the HumanMethylation27 BeadChip array (25,578 probes) interrogated CpG sites within promoter regions and cancer-associated genes, specifically targeting regulatory CpG islands [53]. Next, the HumanMethylation450 array (485,577 probes) interrogated 94% of the 27K canonical CpG sites, spanning diverse regulatory regions including shores, RefSeq genes, FANTOM4 promoters, the MHC region, and enhancers [54]. The latest advancement, the HumanMethylationEPIC v2.0 (EPICv2) BeadChip array, further interrogates over 935,000 CpG sites across biologically relevant regions of the human methylome [55]. The family of Illumina Infinium Methylation BeadChip is widely used across population-based studies for cost-effective, high-throughput, and comprehensive methylation analysis. This technology has been extensively applied in large-scale cancer studies, including TCGA (~8000 profiled samples) and studies within GEO (~ 9000 profiled samples)[41, 56]. Numerous bioinformatics methods and pipelines, such as minfi [57], EpiScanpy [58], EpiMOLAS [59], COHCAP [60], SeSAMe [61], RnBeads [62], and watermelon [63], and Bicycle [64], have been developed to analyze high-throughput methylation data generated by various platforms for epigenome-wide association studies (EWAS).

#### IMPRESS

IMPRESS, a novel multi-cancer detection assay capable of detecting eight cancer types, integrates single-molecule Molecular Inversion Probes (smMIPs) with methylation-sensitive restriction enzyme (MSRE) digestion [46]. MSREs are a class of restriction enzymes that detects and cleave unmethylated CpG sites while leaving methylated sites intact, enabling precise methylation profiling [65]. This technique is built upon earlier restriction enzyme-based methods, such as those using MSREs and methylation-dependent restriction enzymes (MDREs), traditionally used to examine local CpG dinucleotide methylation.

#### HELP assay

The HELP assay is a restriction enzyme-based, high-throughput method that uses ligation-mediated PCR to analyze cytosine methylation by directly representing hypomethylated DNA. Unlike conventional assays, it compares HpaII and MspI digestion profiles to distinguish hypomethylated (HpaII and MspI) from methylated (MspI-only) loci, enabling the precise identification of functionally significant hypomethylated regions, including transcription start sites [66].

A comparative analysis of the profiling methods discussed in the following subsections is presented in Table 2, which summarizes key factors such as genome coverage, cost, advantages, disadvantages, and common procedures for DNA methylation profiling assays.

**Table 2:** Comparison of DNA methylation profiling methods, including genome coverage, cost, advantages, disadvantages, and procedures

DNA methylation profiling type	Procedure	Methods	Cost	Genome coverage	Pros and cons	References
Sequence-based DNA methylation profiling	Bisulphide-based	*RRBS*	$400/sample (prep + sequencing)	~60% promoter, 1.5 million CpGs.	Pros: – Single base resolution for Genome-wide coverage of CpGs. – Dense CpG methylation coverage. Cons: – Restriction enzymes used for preferential selection of sequences at specific sites may cause bias. – Analyzes 10-15% of all CpGs in the genome but lacks the capability to differentiate between 5-methylcytosine (5mC) and 5-hydroxymethylcytosine (5hmC). – Excludes non-CpG regions, genome-wide CpGs, and CpGs without enzyme restriction sites.	[37]
		*TruSeq Methyl Capture EPIC*	$420/sample (prep + sequencing)	~ >3.3 million CpG.	Pros: – Overcomes the limitations of EPIC-array by profiling ~3.74M CpGs at 10X coverage – Reduced cost and time. – Expands on EPIC-array content with by including additional epigenetic regions of interest. – Improved resolution for profiling human methylome by using NGS. Cons: – Limited per-site coverage. – Lower precision compared to arrays	[67]
		*Whole-genome bisulfite sequencing (WGBS)*	~ $1,825/sample (prep + sequencing)	Whole genome (. ~28 million CpG)	Pros: – Methylation of both CpG and non-CpG sites across the entire genome are analyzed at single-base precision. – 5-methylcytosine (5mC) is detected in densely packed, and repetitive regions of the genome. Cons: – Bisulfite treatment converts unmethylated cytosines to thymines, complicating sequence alignment due to reduced complexity. – The conversion of cytosine to thymidine are overlooked during bisulfite conversion – Bisulfite conversion cannot differentiate between 5-methylcytosine (5mC) and 5-hydroxymethylcytosine (5hmC).	[68]
	Open-Chromatin Digestion	*Transposase-accessible chromatin sequencing assay.*	$200 per sample or library preparation and sequencing, separately.	Targets the open chromatin regions.	Pros: – Tagmentation minimizes the DNA input required for ATAC-seq, making it a rapid and highly sensitive assay. – In situ library preparation, and time efficiency of ATAC-seq. Cons: – Technical limitation that can introduce bias. – One bias source is the potential for artifactual tagging of bound chromatin regions during processing.	[69]
	Affinity enrichment based	*MeDIP-seq*	~ $230 per sample for library preparation and $200 per sample for sequencing.	~ 25,000 CpGs sites.	Pros: – Avoids preferential targeting of any specific DNA sequence motif, except for CpGs. – Cost effective. – Gives the whole genome coverage and high sensitivity. Cons: – MeDIP-seq resolution (~ 150 base pairs) is lower than MRE-seq or bisulfite-based methods because the antibody binds to a DNA fragment containing one or more methylated CpGs, making it impossible to determine which specific CpG is responsible.	[70]
Array-based DNA methylation profiling	Bisulphide-based	*Illumina Infinium HumanMethylation BeadChip array*	$250-$300 per sample.	~ 850,000 CpG sites.	Pros: – High-throughput capability – Comprehensive coverage of CpG sites (measures ~ 850,000 cytosines across the genome). – Cost-effective and shows the compatibility with FFPE sample types, allowing for studies on extensive tumor biorepositories. Cons: – Limited resolution and Bias toward certain regions during data analysis. – Increased probe cross-reactivity.	[71, 72]
	Restriction enzymes	*HELP*	–	~ 98.5% CGIs in the human genome	Pros: – Distinguishes the hypomethylated DNA region from the methylated loci. Cons: – A significant limitation is the ambiguous functional interpretation of cytosine methylation in the majority of genomic contexts. In contrast, methylation within CG-rich promoters is a well-established correlate of gene silencing.	[73]
		IMPRESS	Cost effective	Selected 1791 CpG sites which can distinguish tumour and normal samples.	Pros: – High sensitivity and specificity; – Identification of 1791 CpG sites whose DNA methylation patterns differentiate tumor and normal tissue. – Low cost. Cons: – Lacks validation using liquid biopsies – The assay covers only 39% of the methylome's CpG sites due to enzyme recognition limitations. – Cancer type-specific smMIPs for tissue-of-origin determination were not incorporated.	[46]

**a. NGS: Next generation sequencing; b. MeDIP-seq: Methylated DNA immunoprecipitation coupled with next-generation sequencing; c. MRE-seq: methylation-sensitive restriction enzyme sequencing; d. FFPE: Formalin-Fixed, Paraffin-Embedded; e. smMIPs: single molecule Molecular Inversion Probes

### Single-cell methylation assays

Single-cell bisulfite sequencing (scBS-seq) and single-cell reduced representation bisulfite sequencing (scRRBS) provide high-resolution insights into DNA methylation heterogeneity at the individual cell level. These techniques are valuable for studying intra-tumor heterogeneity, enabling the identification of epigenetic variations that contribute to cancer progression and treatment resistance [74].

High-throughput single-cell methylome profiling is advancing through combinatorial indexing, such as the sci-MET (single-cell combinatorial indexing for methylation analysis) method, which employs FANS (fluorescence-activated nuclei sorting) for nuclei isolation, followed by Tn5 tagmentation, PCR for indexing and NGS [75]. Moreover, Chatterton *et al.* 2023 introduced sciEM the first non-bisulfite and enzyme-based single-cell DNA methylation sequencing approach, extending the method of single-cell combinatorial indexing approach (sci) using sodium bisulfite (sciMET) [76]. To address limitations in scalability for large cohorts, blood sample input, and cost-effectiveness, researchers used high-resolution tissue-specific single-cell RNA-sequencing datasets. A scalable DNA methylation atlas for 13 tissues and 40 cell types was validated using bulk and single-nucleus datasets, offering a valuable resource for cancer diagnosis, biomarker discovery, and methylome study interpretation [77]. However, single-cell sequencing faces challenges of high technical noise due to low input material and complex protocols, adversely impacting data reproducibility and reliability, limiting its use for large-scale MCED test applications [78].

## AI Techniques for cancer prediction using DNA methylation

**Fig. 3:**
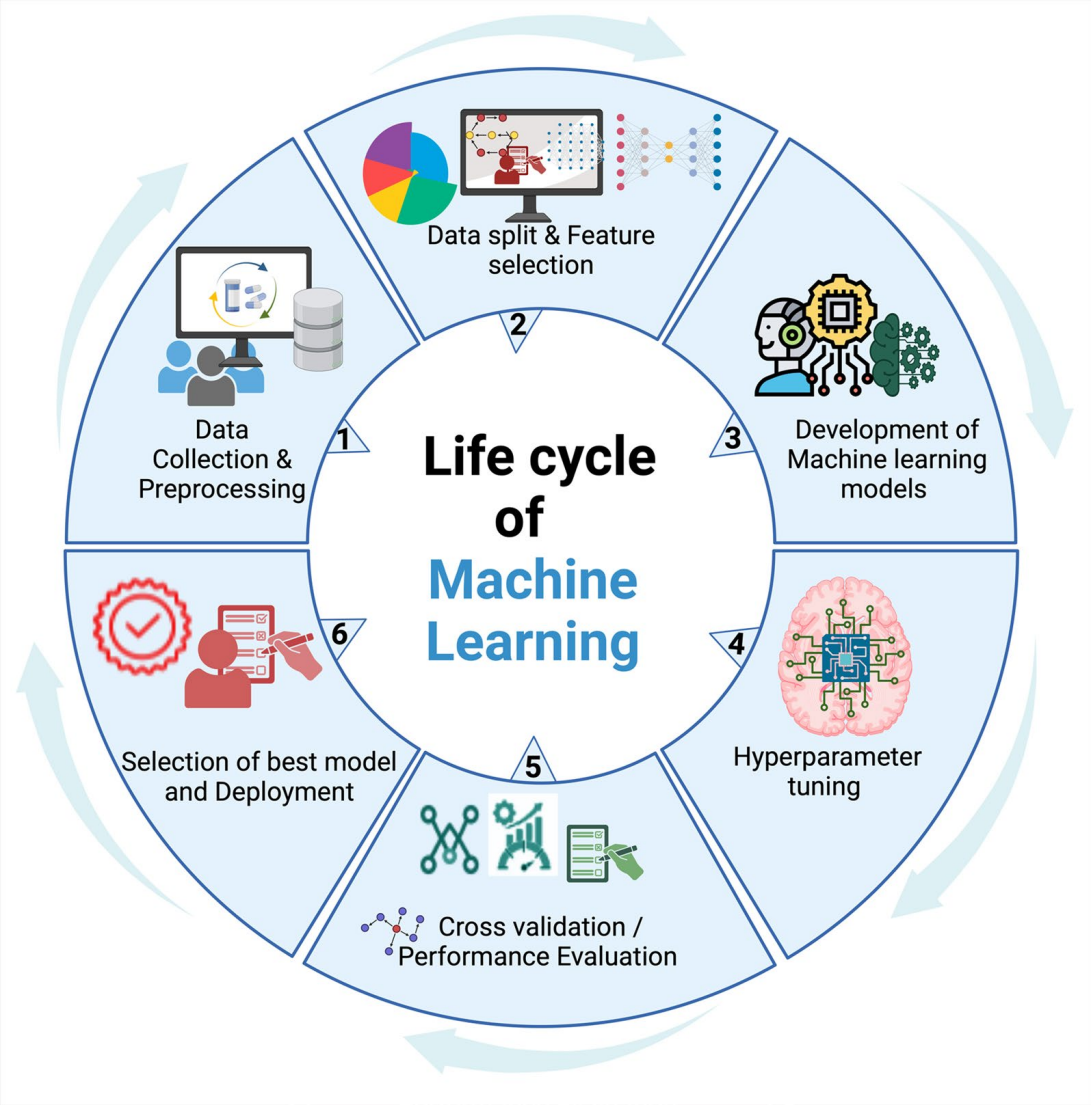
Illustration of the machine learning (ML) lifecycle: The figure depicts key stages of ML lifecycle, represented as interconnected gears to emphasize the iterative nature of the process. It represents the continuous cycle of model development training, assessment, and implementation, illustrating the transition from one phase to the next

AI significantly advances cancer diagnosis and prognosis by enabling high-resolution analysis of imaging, molecular, and clinical datasets. DL and natural language processing (NLP) facilitate early detection, risk stratification, and personalized care. Despite outperforming traditional methods, AI faces challenges in interpretability, data quality, and clinical integration, necessitating multidisciplinary collaboration [79, 80]. Notably, it has been demonstrated that deep neural networks (DNNs) marginally outperformed classical machine learning models in survival prediction achieving 88.58% accuracy compared to 88.51%, underscoring the promise of DNNs in data-driven clinical outcome predictions [81]. Moreover, DL offers a powerful approach for predicting anti-tumor drug combinations by modeling complex biological interactions, addressing drug resistance, and overcoming the limitations of single-agent cancer therapies [82]. Thus, AI algorithms remain integral for developing cutting-edge MCED tests by integrating DNA methylation data with ML and DL algorithms. These AI models enable pan-cancer classification, TOO prediction, and risk stratification, significantly improving the accuracy and efficiency of cancer diagnostics. Supervised and unsupervised learning are fundamental methodologies in ML. Supervised learning relies on labeled datasets, where each input is associated with a known output. In contrast, unsupervised learning deals with unlabeled data, focusing on identifying patterns, structures, or relationships without predefined outcomes. The following section provides a concise overview of the steps involved in pan-cancer classification using ML models, followed by a description of ML methodologies, emphasizing notable research that utilizes these techniques The pan-cancer classification process using ML involves a cyclical workflow consisting of six crucial phases, as illustrated in Fig. 3:

1. Data collection and processing: The first step involves acquiring the DNA methylation datasets. Subsequent steps include, data processing procedures such as normalization, imputation of missing values, adjusting the background, and converting the data for further analysis.
2. Data splitting, data imbalance, and feature selection: This step involves dividing the dataset into training and testing sets while addressing class imbalance issues. The aim is to mitigate bias toward the predominant class and maintain the model's predictive performance. The subsequent step involves selecting the most informative CpG sites using statistical and ML-based feature selection methods (e.g., Lasso, LightGBM).
3. Development of ML models: This step focuses on training multiple models, including tree-based classifiers, deep learning architectures, and probabilistic models to identify and categorize cancer subtypes.
4. Hyperparameter tuning: This step optimizes model parameters through methods like grid search, Bayesian optimization, or genetic algorithms to enhance predictive accuracy.
5. Cross-validation and performance evaluation: This phase ensures models’ generalizability using k-fold cross-validation and evaluates sensitivity, specificity, and area under the receiver operating characteristic curve (AUC-ROC).
6. Model selection and deployment: This final phase involves selecting the best-performing model and deploying it for real-world clinical applications, including MCED tests.

**Fig. 4:**
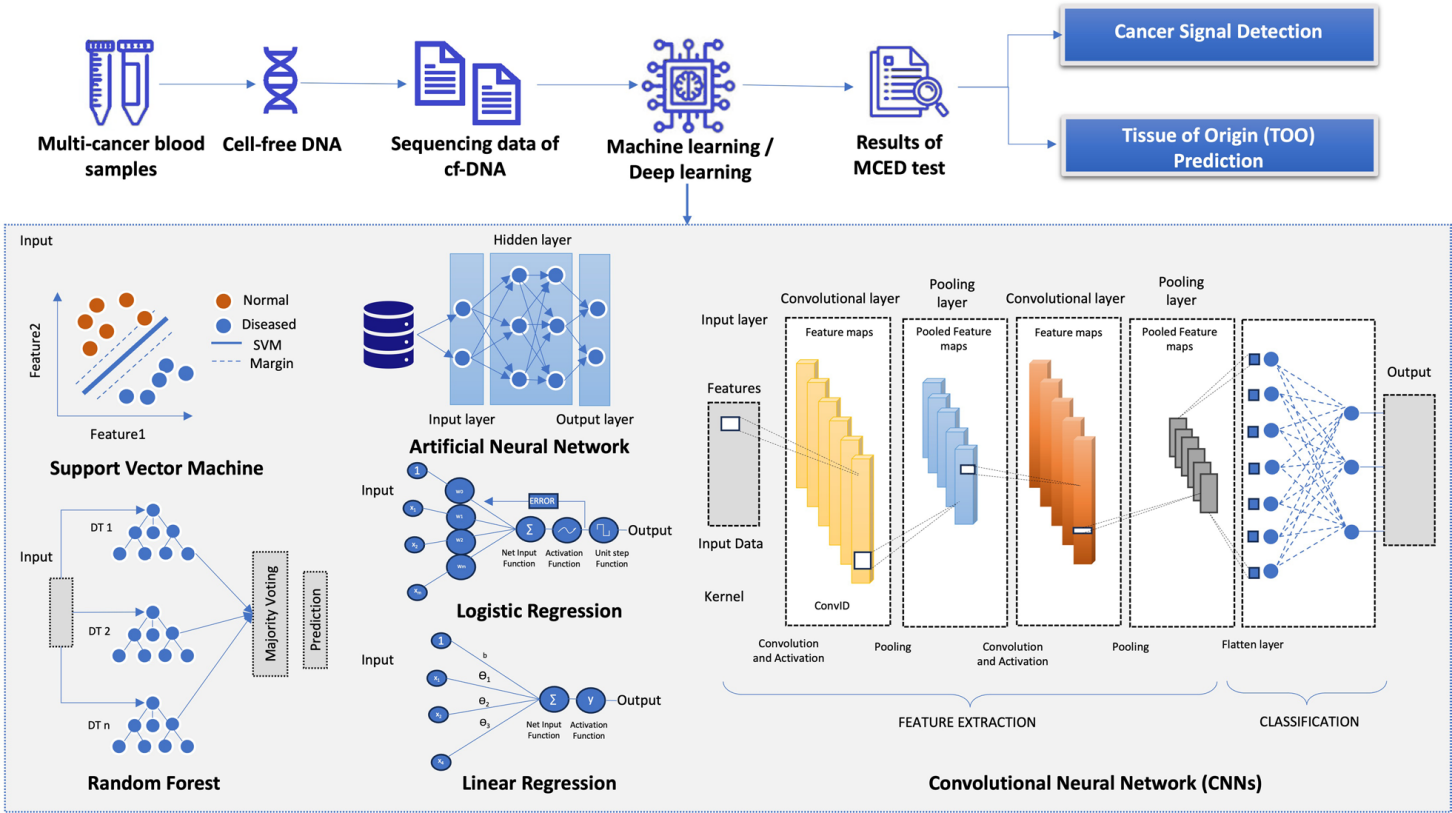
AI-driven Framework for detecting and classification multiple cancer signals: A visual depiction of the AI-based system designed to identify and classify signals from multiple cancers, highlighting the essential components of the machine and deep learning algorithms implemented in these processes

Several ML models have shown outstanding performance in pan-cancer classification using DNA methylation signatures, each employing distinct computational strategies. The following section explores various AI algorithms, focusing on their clinical implications, as illustrated in Fig. 4.

### Machine learning algorithms for DNA methylation-based cancer classification

The characteristics of DNA methylation as a biomarker, when combined with extensive data repositories, enable machine learning algorithms to enhance cancer classification. In this context, ML algorithms, including LASSO regression, logistic regression, and generalized linear models (GLMs), are widely used in DNA methylation-based cancer classification for their ability to detect complex patterns and improve predictive accuracy. Though derived from traditional statistics, these methods meet key criteria for classification as ML algorithms.

i. Data Requirements – ML models, including LASSO and logistic regression, necessitate large datasets to identify intricate DNA methylation patterns and generate accurate predictions [83].
ii. Model Complexity – LASSO regression integrates regularization techniques to effectively manage high-dimensional data and mitigate overfitting [84].
iii. Interpretability – Logistic regression and GLMs offer greater interpretability, with LASSO enhancing this by selecting the most relevant features [85].
iv. Handling Non-Linearity – Logistic regression employs the sigmoid function to model complex input-output relationships, while LASSO and GLMs address multicollinearity by selecting a single variable from highly correlated predictors [86, 87].

### Conventional machine learning algorithms commonly applied in multi-cancer early detection (MCED)

#### Support vector machine (SVM)

SVM is a supervised ML algorithm for classification and regression tasks, designed to construct an optimal hyperplane to separate data points in high-dimensional spaces. Its performance is governed by the hinge loss function, which maximizes the margin between multiple classes [88]. The hyperplane is oriented in the far vicinity from the closest points belonging to each of the classes, known to be as support vectors [89]. The hyperplane equation can be stated as:

$${w}^{T} x+b=0$$

where $w$ is the normal vector, $x$ represents the input feature and, $b$ is the bias term. Its effectiveness in handling the high dimensional genome-wide methylation data, making them suitable for genome-wide methylation studies and MCED tests. In GRAIL's First Circulating Cell-free Genome Atlas (CCGA) Sub-Study, the MCED test was validated on a large-scale population using ML models, including SVM, to analyze cell-free DNA (cfDNA) patterns and classify participants [90].

#### Gradient boosting machines (GBM)

GBMs are ensemble learning algorithms that enhance predictive accuracy by sequentially optimizing weak base learners, typically decision trees, through gradient descent to minimize a specified loss function [91]. The key components include: (i) a loss function to quantify prediction error, (ii) base learners (typically decision trees) built sequentially to address prior errors, and (iii) an additive framework that combines outputs from all learners.

1 $${F}_{0}(x)=\overline{y }$$

2 $${\gamma }_{m}=argmin{\sum_{x\epsilon {R}_{jm}}1/2(yi-\left({F}_{m-1}\left({x}_{i}\right)+ \gamma \right))}^{2}$$

3 $${F}_{m}\left(x\right)={F}_{m-1}\left(x\right)+ \alpha \sum {\gamma }_{mj} 1 ( x \epsilon { R}_{jm})$$

Where $\gamma$ are the predicted values, ${\gamma }_{mj}$ is the sum of all values, ${R}_{jm}$ denotes terminal node, $\alpha$ representing the learning rate, and ${F}_{m}\left(x\right)$ giving the output of the final model. GBMs offer interpretability through tree-based structures and are effective in handling missing data, making them well-suited for complex predictive tasks. In the first CCGA Sub-study, eXtreme Gradient Boosting (XGBoost) was employed as a pan-feature classifier to integrate scores from individual models, with hyperparameters optimized via random search on training data [90]. Nguyen *et al.* 2023 demonstrated that XGBoost’s effectiveness in multimodal plasma cfDNA, integrating methylomics and fragmentomics to distinguish between patients with cancer from healthy individuals and predict TOO. In the concatenated model combining nine features, XGBoost achieved an AUC of 88%, highlighting its robustness in handling complex, high-dimensional data and its strong applicability to MCED testing [92].

#### LASSO regression

LASSO (Least Absolute Shrinkage and Selection Operator) is a supervised regression analysis method that performs regularization and variable selection to improve the prediction accuracy, for both linear and generalized linear models [93]. LASSO demonstrates superior performance due to its lower vulnerability to random errors by setting the coefficients of less important features to zero and eliminating redundant covariates. The regression coefficients in LASSO are estimated using the sparse penalized approaches by optimizing the log-likelihood function while imposing a constraint that the total absolute sum of the regression coefficients, ∑kj=1|βj|, does not exceed a specified positive constant.

$$y= {\beta }_{0}+{\beta }_{1 }{x}_{1}+{\beta }_{2 }{x}_{2}+\dots + {\beta }_{p}{x}_{p}+\epsilon$$

Where y = target dependable variable; ${\beta }_{0}, {\beta }_{1 }, {\beta }_{2 }\dots , {\beta }_{p}$= parameter coefficient for estimation; x_1_, x_2_, x_3_ = independent variables; and $\epsilon$ = error. Further research is needed to evaluate the effectiveness of the LASSO regression algorithm in MCED tests, as its capability for feature selection and handling high-dimensional cfDNA data holds significant promise for enhancing cancer classification and early detection.

#### Logistic regression (LR)

Logistic Regression (LR) is a supervised classification algorithm that models the probability of a binary outcome using the sigmoid function. The finding of optimum results can be defined by applying cost function using gradient methods such as gradient descent and conjugate gradient [94]. Regularization techniques, including L1 (LASSO) and L2 (Ridge), are commonly applied to improve feature selection.

$$y= \frac{{e}^{({b}_{0}+{b}_{1}X)}}{1+ {e}^{({b}_{0}+{b}_{1}X)}}$$

where x= input feature, y = predicted value, ${b}_{0}$ = bias; ${b}_{1}$ = input coefficient. Moreover, LR is used to form an Ensemble model which involves the use of a stacking ensemble model with logistic regression to integrate the predictions from individual feature models, achieving AUC of 93% [92]. Infact, LR outperformed several other algorithms—including k-NN, Random Forest, and SVM—with an AUC of 0.96. The latter demonstrated exceptional performance in cfDNA-based multimodal cancer classification and prognostic assessment [95].

### Multinomial logistic regression (MLR)

Multinomial Logistic Regression (MLR) extends binary logistic regression to model categorical outcomes with more than two classes. The latter is done by estimating the probability of each class based on the log-odds transformation (logit), according to the equation:

$$Log \left(odds\right)=logit\left(P\right)=\text{ln}\left({P}_{1}-P\right)=a+{b}_{1}{x}_{1}+{b}_{2}{x}_{2}+{b}_{3}{x}_{3}$$+ …

Where $P$ represents the likelihood of a case belonging to a specific category; exp denotes the exponential value (~ 2.72); $a$ being the constant of the equation, and $b$ represents the coefficient of the predictor or independent variables [96]. In the CCGA Sub-Study, MLR played a key role in predicting Cancer Signal Origin (CSO) labels by analysing fragmentomic patterns in WGBS-based methylation classifiers, gene disruptions in SNV-WBC classifiers, and read depth variations in WGS-based SCNA classifiers, thereby enhancing tumor origin identification in MCED tests [90].

#### Random forests (RF)

Random Forests (RF) are supervised, non-parametric, tree-based ensemble approaches that construct multiple decision trees during training. It determines the final output by selecting the most frequent class for classification or averaging the predictions for regression. This algorithm is widely used for feature selection and classification, exhibiting higher performance accuracy than SVM, Decision Tree, Multilayer Perceptron, and K-Nearest Neighbors [41]. It combines the principles of adaptive nearest neighbors with bagging, enabling efficient data-adaptive inference. The greedy nature of the algorithms optimally splits the trees at each step while applying regularization for effective complex data and managing feature interactions and correlations. The SelectFromModel function with a 0.0001 threshold was employed for feature selection in the RF model for classifying different cancer types. GridSearchCV was used for hyperparameter selection, and the model was further validated using k-fold cross-validation [92]. Also, the RF model was utilized for detecting cancer and classifying the TOO, incorporating rigorous cross-validation and feature selection techniques[97]. Moreover, Zhang *et al* 2024, used Random Forest with feature selection on serum microRNAs to help predict the tissue of origin in 13 cancer types, achieving up to 95% accuracy in the top 3 predictions—supporting its use alongside MCED screening [98].

#### Generalized linear model (GLM)

The Generalized Linear Model** (**GLM) is a class of supervised regression models used to describe relationships between one or more predictor variables and a response variable. GLM is designed to handle diverse error distributions and allow for flexible and non-linear feature correlations by using a separate underlying statistical distribution. Bao *et al.* 2022, utilized GLM for the construction of the ensemble learning base model, incorporating other algorithms such as GBM, Random Forest, Deep Learning, and XGBoost. The base model predictions were aggregated into a large matrix, which was then utilized to train the final stacked ensemble model. The researchers assessed the cancer detection model on a test dataset and validated the cancer origin model using true-positive cases [99].

#### k-Nearest neighbors (kNN)

The traditional K-Nearest Neighbors (KNN) algorithm is a supervised, non-parametric method primarily used for classification by comparing a sample to its closest neighbors within the feature space. It predicts the label of a query point based on the majority class (for classification) or average value (for regression) of its *k* closest training samples, using distance metrics like Euclidean or Manhattan distances [100]. Upon identifying the k nearest data points, the algorithm employs a majority voting mechanism to ascertain the most frequently occurring class among these neighbors. The classification accuracy of the algorithm is highly dependent on the number of *k,* necessitating testing of different values to determine the optimal one for the dataset [101].

$$\widehat{y}=f\left(x\right)=\frac{1}{k}\sum_{i \in {N}_{k}}{y}_{i}$$

$\widehat{y}$ denotes the estimated continuous value for the given query point *x*; *k* represents the total number of nearest neighbors used for prediction; *y*_*i*_ represents the actual target value of the *i*^*th*^ neighbor; *N*_*k*_ signifies the collection of the *k* nearest neighbors to *x*; $\frac{1}{k}\sum_{i \in {N}_{k}}{y}_{i}$ computes the average target value of these *k* selected neighbors. In recent applications, such as demonstrated by Hajjar, M. *et al*. 2024, KNN was tested in cfDNA-based cancer detection, but Logistic Regression ultimately outperformed it in sensitivity within a multimodal diagnostic approach (cfDNA fragmentomic and genomic features)[90].

### Deep learning for cancer methylation analysis

Deep learning uncovers complex structures within large datasets using the backpropagation algorithm, which optimizes its internal parameters to compute representations in each layer based on the previous one. Common deep learning algorithms, such as convolutional neural network (CNN) and graph convolutional neural network (GCNN), are widely used for classification of tumor of origin prediction models. These neural networks are the amalgamation of interconnected nodes or neurons that process and learn from the training data.

#### Graph convolutional neural network (GCNN)

Graph Convolutional Neural Networks (GCNNs) classifies TOO in multi-cancer detection by utilizing graph-structured relationships among cancer types. These models analyze input graphs where patients are nodes and similarities are edges, often constructed using the k-nearest neighbors (k-NN) algorithm. Applying GCNNs to ctDNA-based detection is challenging due to ctDNA's low abundance and variability, impacting model reliability [102, 103]. To address this, Nguyen *et al.* 2023 introduced SPOT-MAS, a multimodal assay combining methylomics, fragmentomics, copy number variations, and end motifs using shallow genome-wide sequencing (~0.55×). The resulting machine learning method achieved 72.4% sensitivity at 97.0% specificity, with a tumor-of-origin classification of five cancer types reaching 0.7 accuracy. Although this shows a promising potential of ctDNA-based assays, data sparsity remains a constraint for graph-based learning models. To enhance feature selection in GCNN applications, the authors used importance scores (*Fi*), with a cutoff δf = 0.9 to minimize noise from low-abundance ctDNA signals and improve classification accuracy [92].

#### Neural network-based machine learning framework

Machine learning frameworks based on neural networks are widely known for their ability to perform robust predictions across various cancer data types and identify potential biomarkers. For instance, EMethylNET *(Explainable Methylome Neural network for Evaluation of Tumours),* is a hybrid model integrating XGBoost and a deep neural network for multiclass and binary classification of DNA methylation microarray data. This framework was applied to the dataset from 13 cancer types and corresponding normal tissues collected from TCGA. EMethylNET utilized an XGBoost model with 800 estimators, a maximum tree depth of three, and a tuned learning rate for optimal performance. To prevent overfitting, 50% of features and samples were randomly selected for each tree and only features with a positive importance score in XGBoost were used as input for the feedforward neural network. The neural network was trained with the Adam optimizer and cross-entropy loss, using a “Talos-based hyperparameter search” with 30% validation data and early stopping for selecting the best model within 500 epochs [104]. Moreover, CrossNN is another a machine learning framework based on neural networks that accurately classify tumor types using DNA methylation profiles from various platforms, regardless of epigenome coverage and sequencing depth. Feature selection involved encoding methylated/unmethylated probes and filtering uninformative probes. The neural network model was trained using reference methylomes dataset, with beta values binarized at a threshold of 0.623 and zero variance features removed. To maximize feature utilization, a fixed sample rate was employed, with random masking of 0.25% of training samples, determined via 5-fold cross-validation. A normalization function and a SoftMax layer converted outputs into probabilities of brain tumor subtypes, and the model using PyTorch 1.13.0 was developed using the Adam Optimization Algorithm [105].

Here, we provide a concise and updated comparative summary of key artificial intelligence algorithms currently applied in multi-cancer early detection (MCED) using cfDNA, highlighting their core principles, applications, advantages, and limitations (Table 3).

**Table 3:** Summary of key AI/ML methods used in cfDNA-based multi-cancer early detection (MCED), highlighting their core principles, applications, strengths, and limitations

S.no	Method	Key characteristic	MCED application	Pros	Cons	Refs.
1.	Support Vector Machine (SVM)	Constructs optimal hyperplanes to separate classes in high-dimensional space using hinge loss.	Analyzing cfDNA patterns to classify cancer types.	Effective in high-dimensional genomic data; suitable for genome-wide methylation studies.	Less scalable with large datasets; sensitive to kernel selection	[90]
2.	Gradient Boosting Machines (GBM/XGBoost)	Sequentially builds trees to minimize prediction error using gradient descent.	Multimodal cfDNA classification, integrating methylomics and fragmentomics.	Handles missing data well; interpretable; high predictive power	Computationally expensive; risk of overfitting if not tuned properly	[92]
3.	LASSO Regression	Performs regularization and feature selection by shrinking coefficients to zero.	High-dimensional cfDNA feature selection and cancer classification.	Reduces overfitting; useful for sparse data.	Can exclude relevant features; linear model assumption	[93]
4.	Logistic Regression (LR)	Models probability of binary/categorical outcomes using sigmoid/logit functions.	Multimodal cancer classification, CSO prediction.	Simple and interpretable; performs well on structured cfDNA data.	Limited in non-linear data separation; sensitive to multi-collinearity	[95]
5.	Random Forest (RF)	Ensemble of decision trees using bagging for robust classification.	TOO prediction using cfDNA and microRNA data.	Handles high-dimensional data well; good for feature selection	Less interpretable; slower for large data	[98]
6.	Generalized Linear Model (GLM)	Extends linear regression to allow response variables with different distributions.	Base model in ensemble learning for cancer detection.	Flexible for various distributions; interpretable	Limited to linear relationships; less powerful in complex data	[99]
7.	k-Nearest Neighbors (kNN)	Instance-based learning relying on distance from nearest neighbors.	Tested for cfDNA cancer detection in multimodal setups.	Simple and intuitive; no training phase	Sensitive to noise and k-value; poor scalability	[90]
8.	Graph Convolutional Neural Networks (GCNN)	Uses graph-structured data for relational learning between samples.	TOO classification using ctDNA-based multimodal data.	Captures complex inter-sample relationships;	Sensitive to ctDNA sparsity; data quality dependent	[92]
9.	Neural Network-Based Framework (EMethylNET, CrossNN)	Deep learning models combining neural networks with feature engineering.	DNA methylation-based tumor classification.	High accuracy; captures non-linear patterns; scalable	Requires large data and tuning; low interpretability	[104, 105]

### Interactive machine learning (IML) algorithms

In the domain of cancer epigenomics, where data are inherently high-dimensional, sparse, and biologically complex, interactive machine learning (IML) has emerged as a promising paradigm to bridge gaps left by conventional machine learning methods that struggle with small or noisy datasets [106]. IML technique, which involves the human-in-the-loop strategies drawn from active learning, Explainable AI, and reinforcement learning has enabled iterative and expert-guided model refinement [107–109]. These approaches have shown tangible benefits in epigenetic signature interpretation, feature selection, and model explanation, which are crucial tasks in the analysis of DNA methylation patterns and multi-omics data for cancer detection. For example, in the context of early cancer screening and minimal residual disease detection, IML frameworks have been used to identify differentially methylated regions (DMRs) by reducing the annotation burden through active learning, and to guide expert-driven decision-making in feature prioritization [110]. Recent developments even explore human-in-the-loop reinforcement learning models that integrate clinician expertise in selecting biologically meaningful features, enhancing model robustness in pan-cancer classification and tissue-of-origin prediction [111]. These developments illustrate the transformative potential of IML to not only handle the complexities of cancer epigenomics but also accelerate the clinical adoption of AI systems by embedding expert knowledge into every stage of model development [112]. Thus, the integration of IML into epigenetics offers a fertile ground for advancing explainable, accurate, and patient-aligned machine learning tools for cancer diagnostics and therapeutics.

## Multi-cancer early detection (MCED): pipelines, technologies and industry advancement

Recent advancements in detecting cancer-related changes in ctDNA and other liquid biopsy biomarkers have facilitated the development of MCED tests. These tests consolidates the detection of multiple low-prevalence cancers into a single diagnostic tool, improving positive predictive value (PPV) through high specificity while reducing the need for invasive screening procedures. Also, MCED analyzes a broad range of biological markers, including tumor cells, DNA, RNA, proteins, and other molecules [113]. cfDNA being the primary analyte, is analyzed using WGS is used for identifying somatic copy number alterations in the DNA sequence, including fragment endpoints, fragment length, and allelic imbalance [90]. Some of the key features ensuring MCED test accuracy and clinical utility include: 1) case-control efficiency for initial development and validation, despite potential spectrum bias; 2) varying sensitivity and specificity across cancer types, stages, and study designs; and 3) essential clinical validation studies at the population level to ensure effectiveness [13].

**Fig. 5:**
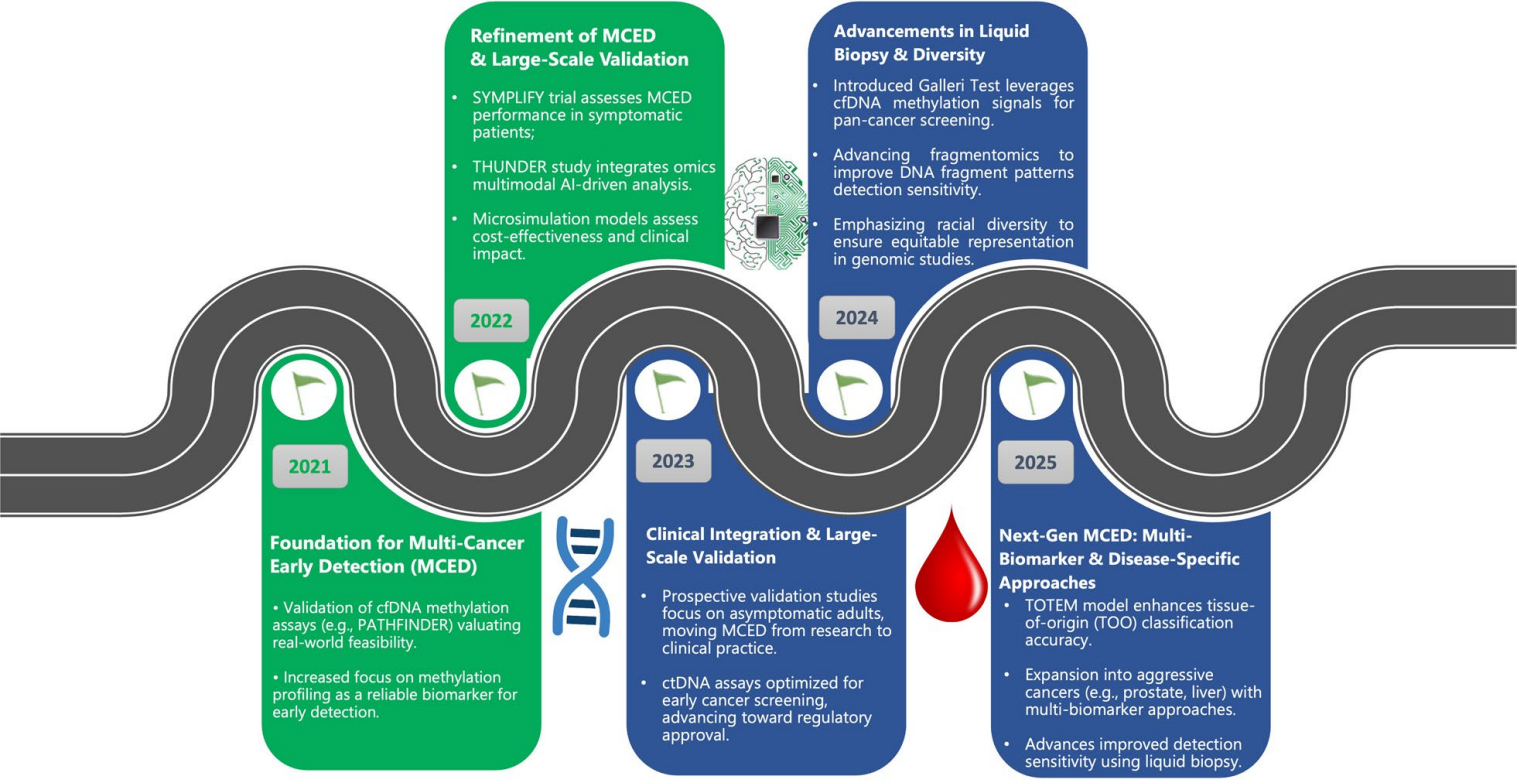
Timeline depicting advancements in Multi-Cancer Early Detection (MCED) from 2021 to 2025, highlighting progress in liquid biopsy, multi-omics integration, validation studies, and clinical implementation for early cancer screening

The integration of MCED techniques into clinical practice demands a careful evaluation of early detection benefits, such as decreased mortality, against the risks associated with false positives, overdiagnosis, and unnecessary treatment [114]. To elucidate the progression of these advancements, we performed a systematic PubMed search employing the terms (MCED OR "multi-cancer early detection") AND (methylation OR "DNA methylation"), which uncovered crucial milestones in MCED evolution. Figure 5 illustrates this developmental trajectory, showcasing landmark achievements, including the clinical implementation of methylation-based diagnostic tools. The subsequent sections will explore more elaborate MCED workflows and recent innovations in detail.

### Clinical pipelines and industry advancements in MCED technologies

In recent years, the development of MCED tests has surged, driven by advances in sequencing technologies and bioinformatics workflows. Several biotech companies, including GRAIL, are at the forefront of MCED innovation, using ctDNA methylation analysis to detect over 50 types of cancer. One of GRAIL’s initial studies, the CCGA by Liu *et al.* 2018, demonstrated that WGBS outperforms targeted mutational panels and WGS in cancer detection. This analysis involved 2402 samples (both controls and newly diagnosed untreated cancers across 20 types) using sequencing assays such as cfDNA/white blood cell (WBC) targeted sequencing, WGS, and WGBS, with cancer-specific sensitivities ranging from 54% to 94% [115]. GRAIL’s Galleri test, validated in 4077 samples, achieves 51.5% sensitivity (stage I–IV) and 88.7% TOO accuracy [116, 117], with an estimated PPV minimum of 84.2% [118]. Another cfDNA-based probabilistic method, CancerLocator, detects cancer and predicts TOO using genome-wide DNA methylation data. This method outperforms traditional multiclass classification measures on both simulated and real data by selecting CpG cluster with high methylation levels and applying mixture modeling, maximum-likelihood estimation, and Pearson’s correlation [119]. Moreover, the cfMeDIP-seq library, consisting of 189 plasma samples from seven types of cancer, was used to identify Differentially methylated regions (DMRs), which were then applied to construct highly accurate cancer-specific classifiers [120]. Additionally, a targeted bisulphide sequence-based methylation assay analyzing 9223 hypermethylated CpG sites in cfDNA accurately detected, classified, and differentiated various advanced cancers, identifying over 80% of cases across 32 common cancer types [121]. The development of multi-biomarker based MCED test may also enhance the ability to detect molecular and phenotypic tumor heterogeneity. For instance, an optimized and validated novel three-marker methylation-based blood test model designed by Funderburk K *et al*. 2023 using *TLX1*, GALR1, and *ZNF154* markers in array-based methylation data demonstrated superior sensitivity, specificity, and PPV across 14 cancer types. This study also employed logistic regression models for each cancer types [122]. Similarly, CancerSEEK, which integrates mutation and protein biomarkers, demonstrated 62% sensitivity across eight types of cancers [123]. Table 4 summarizes the key features and performance of widely used DNA methylation-based MCED tests, identified from literature surveys, companies’ websites, and conference abstracts.

**Table 4:** Summary of DNA methylation-based MCED tests: key features and performance metrics from literature and industry sources

Company/ Method	Technology	Cancer types Detected	Sensitivity/Specificity/TOO accuracy	Sample Type	AI algorithm	Year/Country	Features	Refs.
IvyGene	Whole genome Methylation Analysis using PCR & NGS	Liver, breast, colorectal, and lung cancers	84%; 90%; N/A	Plasma (ctDNA)	Utilizing AI, combined with cutting edge technology for analysing ctDNA methylation patterns.	USA 2018–2019.	- Offers a non-invasive solution for early cancer detection. - Measures the status of the methylation levels of cfDNA.	[124]
cfMeDIP-Seq	cfDNA Methylated DNA Immunoprecipitation Sequencing	Pancreatic, colorectal, breast, lung, renal, bladder, AML, glioma	AUROC: 0.980 (AML), 0.918 (PDAC), 0.971 (LUC); High; N/A	Plasma (cfDNA)	Incorporates ML (Random Forest) classifiers on cell-free DNA methylation.	Canada; first major publication in 2019– 2020	- Enriches for methylated DNA without bisulfite conversion. - Demonstrates high sensitivity to low fractions of cancer DNA. - Effective for subtype-specific and early-stage cancer detection.	[125, 126]
PanSEER	ctDNA-Methylation Biomarkers & PCR Sequencing	Colorectal, esophageal, liver, lung, and stomach cancers	88.2% (post-diagnosis);95%; N/A	Plasma (cfDNA)	Utilizes a ML algorithm (logistic regression classifier) trained on methylation markers for early detection and TOO prediction.	China/USA collaboration 2020	- Analyzes 477 cancer-specific DMRs with 10,613 CpGs, - Offers high sensitivity down to 0.01% cancer DNA. - Cost-effective with low cfDNA input.	[127]
Galleri	Targeted DNA Methylation Sequencing	Over 50 cancer types	51.5% overall; 16.8% (stage I), 40.4% (stage II), 77.0% (stage III), 90.1% (stage IV); 99.1%; 88.7%.	Plasma (cfDNA)	Advanced ML on targeted methylation data to detect and localize TOO of multiple cancers from cfDNA.	USA; 2018.	- High specificity for low tumor fraction. - Variable sensitivity by cancer type and stage. - Validated in CCGA and PATHFINDER trials.	[128]
CancerSEEK/Exact Science (USA)	Mutation and Protein Biomarker Analysis; Liquid Biopsy using cfDNA and ML for cancer prediction	About 8 cancer types (ovary, liver, stomach)	62% (1005 as number of patient with cancer); 99.1%; 63%.	Plasma (ctDNA)	ML classifier combining ctDNA mutations and selected protein biomarkers.	USA; 2019–2020.	- Integrates multi-omics. - Detects cancers without standard screening.	[123, 129]
Adela Bio/Adela	Cell-free methylated DNA immunoprecipitation-sequencing.	About 7 cancer types	70%–75%; 99%; 91%.	Plasma (cfDNA)	ML application analyzing methylome signals	USA; 2021	- Avoids bisulfite conversion; - Effective for early-stage and subtype-specific detection; - Utilizes ML algorithms to identify key methylated regions in cfDNA.	[130]
TR(ACE) /Biological dynamics	Alternating current electrokinetics (ACE) platform to purify extracellular vesicles from plasma; ML algorithm.	About 7 cancer types	Sensitivity of 71.2% (95% CI: 63.2–78.1); 99.5% ; 43.8% - 95.5%	Circulating extracellular vehicles (EVs)	Uses ML classifier on extracellular vesicles (EV)–associated biomarkers.	USA; 2020–2021	- Utilizes ACE platform to isolate circulating EVs from plasma and is used for multi-marker analysis. - Efficient analysis of a large number of samples.	[131]
Cancer detector	cfDNA bisulfite sequencing, probabilistic model.	Study report on liver cancer but claims to detect all types of cancers.	94.8%; 100%; N/A.	Plasma (cfDNA)	NA	USA; 2018	- Developed CancerDetector which focuses on joint methylation states improves sensitivity for detecting abnormal cfDNAs. - Achieves high accuracy and its prediction is consistent with clinical information.	[132]
EpiPanGI Dx	Bisulfite sequencing method and Machine learning	GI cancers (CRC, pancreatic, stomach)	AUC: 0.88; 96%; 0.85–0.95.	Plasma (cfDNA)	Methylation-based biomarker approach with machine learning for GI cancer detection.	U.S., Germany, Japan, South Africa, and Spain; 2020–2021.	- This test identified the three distinct DMR panels that are Cancer-Specific Biomarker Panels. - Pan-GI Cancer Panel and multi-cancer TOO prediction panel.	[133]
Burning Rock Dx	Targeted methylation sequencing assay combined with machine learning.	6 Cancers (liver, colon/rectum, esophagus, pancreas, lung and ovary)	80.6%; 98.3%; 81.0%.	Plasma (cfDNA)	- ML approaches (SVM) for ctDNA methylation or targeted gene panel detection. - Multi-class logistic regression was used to predict tissue origin.	China; 2020	- Optimized for low-depth sequencing; validated in THUNDER-II trial. - Shows high specificity and accurate TOO prediction.	[134]
GENECAST	Targeted methylation sequencing	14 cancer types	72.86% ; 96.67% (AUC = 0.86); N/A.	Plasma (cfDNA)	N/A	China; 2019–2021	- The model developed was based on 37 MCB. Biomarker methylation differences were computed using a HM-score.	[135]
Guardant Reveal	Methylation panel (500 CpGs) + fragmentomics + ML	13 cancers	76.4%/42%; 97.9%; 82%.	ctDNA	Statistical and ML–based algorithm for ctDNA signals (both genomic, epigenomic and fragmentomics).	Usa; 2021	- Combines methylation and fragment size analysis. - FDA-approved for colorectal cancer recurrence.	[136]
DELFI (Delfi DX)	Genome-wide cfDNA fragmentation + ML	7 cancers (lung, breast, liver)	73% (Stage I–II); 98%; 85%.	Plasma (cfDNA)	ML on cfDNA fragmentomics (fragmentation profiles).	USA; 2019	- Low-cost WGS approach. - Detects fragmentation patterns linked to chromatin instability.	[137]
OncoSeek	Protein biomarkers (CA-125, CEA) + AI	9 cancers	51.7%; 95%; 66.8%.	Plasma (serum)	Uses AI for calculating the POC index	California, USA;	- Low-cost protein-based test. - Integrates clinical metadata for risk stratification.	[138]
SPOT-MAS (Gene Solutions)	Targeted methylation (14 genes) + ML	5 cancers (breast, liver, CRC, lung)	78%; 99.8%; 84%	ctDNA	Integrates ML analysis on ctDNA mutation and methylation signals.	Vietnam; 2021–2022	- Validated in 10,000+ Vietnamese patients. - Optimized for low-resource settings.	[139, 140]
SeekInCare (SeekIn)	Methylation + CNVs + ML	20 cancers (NSCLC, CRC, liver)	65.5%; 97%; 93%	Plasma (cfDNA)	Deals with Multi-omic (ctDNA + protein) data; gradient-boosting machine ML algorithms for early detection and surveillance.	China; 2020–2021.	- Resource-optimized cancer screening for large populations.	[141]
Freenome	Methylation + fragmentomics + proteomics + gradient boosting	8 cancers (CRC, lung, breast)	79.2%; 92% in CRC; N/A.	Plasma (cfDNA)	ML enabled multi-omics	USA; 2019	- Multi-omics approach; - This is under clinical evaluation in PROSPECT study (NCT05581476).	[142]

*a. ACE: alternating current electrokinetics, b. EV: Extracellular vesicles, c. MCB: methylation-correlated blocks, d. HM score: Hypermethylation score; e. WGS: Whole genome sequencing. f. SVM: Support Vector Machine., g. POC: probability of cancer; h. ML: Machine learning, i. AI: Artificial Intelligence.; f. N/A- Not available

### Machine learning driven pan-cancer classification pipelines

Recent progress in high-throughput technologies has been crucial developing MCED tests. Nevertheless, these technologies alone are not sufficient for precise and scalable cancer diagnostics. Machine learning (ML) has emerged as a critical tool in improving the accuracy of MCED tests, facilitating robust pan-cancer classification. In the following section, we discuss key algorithms that have been applied for enhanced pan-cancer detection:

- XGBoost: Cui, P. *et al.* 2024 developed an XGBoost model using sequenced methylation data from WGS and WGBS of cfDNA obtained from patients with cancer and healthy controls. The model was trained on a numerical matrix of 11-nt cleavage windows and their corresponding values, effectively distinguishing between hyper- and hypo-methylated CpG sites. It achieved AUCs of 0.959, 0.896, and 0.827 for HCC, lung cancer, and colorectal cancer, respectively [143].
- Random forest: Modhukur *et al.,* 2021 demonstrated that Random Forest outperformed other algorithms like SVM, Naive Bayes, and XGBoost in classifying cancer types based on TOO prediction. This model achieved an average accuracy of 99% highlighting its robustness in distinguishing cancer types based on methylation profiles [88].
- MetDecode: MetDecode**,** a CNN-based tool, achieved 84.2% accuracy of TOO using whole-genome methylation data. By leveraging DNA methylation signatures from integrated in-house and public whole genome methylation datasets, this approach demonstrated strong performance in identifying the TOO in cfDNA. Specifically, it achieved a limit of detection (LOD) of 2.88% with Pearson correlation coefficients exceeding 0.95, outperforming similar TOO prediction methods like CancerLocator and CelFiE [144].
- TOTEM (cTdna Origin Tracker dependent on Epigenetic Methylation markers): This algorithm is being used for MCED test and cancer signal origin (CSO) localization, based on enzymatic conversion-based targeted methylation sequencing of patient samples. The model achieved AUC values of 0.907, 0.908, and 0.868 in the training, testing, and independent validation cohorts, respectively, with specificities of 98%, 100%, and 98.6%. The model’s robustness was further validated using a smaller set of 21 diagnostic markers and 214 cancer signal origin (CSO) markers, yielding a testing AUC of 0.866 and a top-2 accuracy of 83.1% [145].
- Methylation-based classifier (MFCUP): Sun M. *et al.,* 2024 developed a novel methylation-based classifier (MFCUP) to predict the tissue of origin in CUP patients. Leveraging a large methylation dataset of 32 cancer types, the researchers trained a ML model with random forest for feature selection and elastic net for classification. This approach significantly improved accuracy from 84.8% to 93.4% on Infinium EPIC and 450K array while enhancing the sensitivity (0.8 to 1) and specificity (0.995 to 1) across 25 different cancer types [146].
- Microsimulation model: A recently developed microsimulation model assessed the performance of the Galleri^Ⓡ^ MCED test in cancer screening trials, presenting a range of positive predictive value (PPV) values from 48% to 61%. After three annual screenings, early-stage 23 different cancer detection (stage I/II) increased by 9% to 14%, incidence of stage-IV cancers decreased by 37% to 46%, and mortality rate reduced by 13% to 16% [147].

### Deep learning approaches for pan-cancer classification pipelines

Traditional screening methods analyzing blood samples under microscopy is time-consuming, prone to bias, and dependent on the expert availability. In contrast, deep learning algorithms offer automated, and efficient solutions, enabling tumor detection from large-scale digital histopathology images with improved accuracy using CNN [148].

- Convolutional neural networks (CNNs): Convolutional neural networks (CNN) are the type of neural network capable of discerning distinctive patterns and characteristics associated with diverse forms of cancer using image-based and gene expression datasets. The CNN model comprises multiple layers, including the input layer, convolutional layer, and pooling layer, enabling hierarchical feature extraction for creating the data models. Utilizing a one-dimensional kernel with two input vectors as its foundation, CNNs can effectively predict cancer types [149].
  Several studies showcase an architecture of the CNN for the classification of cancer epigenetics and diseases. For instance, iCancer-pred [150] leverages DNA methylation data for cancer diagnosis through a two-stage feature selection process using the coefficient of variation and elastic network techniques. iCancer-pred incorporates fully connected neural networks for binary (sigmoid) and multiclass (softmax) classification, achieving high accuracy (98.37%) and AUC (99.68%) in distinguishing cancer subtypes. Similarly, DISMIR utilizes a CNN-based model and introduces the ‘switching region’ feature to identify cancer-specific differentially methylated regions, enhancing cancer signal detection at read resolution for highly sensitive plasma-based cancer diagnostics [151]. Although CNN has not yet been fully utilized in MCED tests, it presents significant potential for identifying methylation biomarkers crucial for early cancer detection and facilitating accurate pan-cancer classification.
- Variational autoencoder (VAE): A variational autoencoder (VAE) is a generative neural network comprising an encoder and a decoder for efficient feature learning. Such methods have been increasingly applied to epigenetic cancer subtype classification using multi-omics datasets, gaining attention for pan-cancer prediction. For instance, OmiEmbed, developed by Zhang *et al.* 2021, leverages a variational autoencoder (VAE) to encode high-dimensional multi-omic data into a compact latent space. A multi-layer fully connected network then processes this representation for tumor classification, primary site identification, and disease stage prediction. This method outperformed traditional machine learning models, achieving an AUC ROC of 0.9943 versus 0.9863, highlighting its efficacy in multi-cancer classification and survival analysis [152]. Next, Methylnet, a pretrained variational autoencoder (VAE), was utilized for feature extraction in multi-output regression and classification tasks, including pan-cancer subtypes and smoking prediction. Optimized via autonomous hyperparameter scanning, it employed Shapley Feature Attribution to identify key CpGs, achieving 97% accuracy, precision, sensitivity, and F1 score in pan-cancer classification [153]. Also, MetaCancer is a DL model developed for pan-cancer metastasis prediction that integrates TCGA multi-omics data and employs a convolutional variational autoencoder for feature extraction, followed by a fully connected network for classification. MetaCancer outperformed the SVM ensemble, achieving 88.85% accuracy versus 82.50% [154].
- Graph convolutional neural networks (GCNNs): Four innovative GCNN models utilize unstructured gene expression data to classify samples into 33 cancer types or as normal tissue. Validated on the TCGA dataset, GCNNs achieved over 94% accuracy, demonstrating their potential utility in cancer diagnosis [155]. Its architecture comprises an input graph encoded by an adjacency matrix, followed by graph convolutional layers that perform coarsening and pooling. A hidden layer is subsequently linked to a fully connected softmax output layer for classification. Moreover, Categorical cross-entropy was utilized as the loss function, with the Adam optimizer applied across all four GCNN models. Optimal hyperparameters, including pooling strategy, learning rate, hidden layer size, and batch size, were identified through Random Search [156].
- CancerNet: CancerNet utilizes a deep learning architecture to analyze methylation data for cancer detection. It comprises an encoder with two dense layers (ReLU activation), a probabilistic layer, a classifier (ReLU and softmax layers), and a decoder (ReLU and sigmoid layers). This model accurately classifies 33 cancer types with >99% F-measure, distinguishing primary, metastatic, and pre-cancerous lesions [157].

### Prognostic insights into MCED tests in patient management

MCED tests provide valuable prognostic insights for patient management, by considering cancer subtypes and detection timing, critically impacting patient management strategy. Xiaoji Chen *et al.* 2021, demonstrated that cancers undetected by the MCED test had better survival rates over three years compared to the detected ones. The finding holds true regardless of their clinical stage, underscoring the potential correlation between test detection and tumor fraction in cfDNA [158]. Moreover, the prognostic implication of the MCED test depends on histological subtype and detection timing. The findings suggest MCED test do not predict relapse within five years post-resection and an increased rate of pathological upstaging [159]. Moreover, Hubbell E *et al.* 2021 developed an interception model integrating Surveillance, Epidemiology, and End Results (SEER) data with MCED test to improve late-stage cancer prognosis. From their projection, MCED test could intercept 485 cancers per 100,000 annually, reduce late-stage incidence by 78%, and lower 5-year cancer mortality by 39%. The latter corresponds to 104 fewer deaths per 100,000 or a 26% reduction in overall cancer-related mortality [160]. Certainly, the tests also exhibit moderate sensitivity with robust detection of clinically aggressive cancers while often missing indolent or early-stage tumors [161, 162].

### Validation of MCED tests in symptomatic patient cohorts

The prospective evaluation of the targeted methylation-based MCED test in a large cohort of symptomatic patients supports its effectiveness in aiding clinicians with urgent decision-making and optimizing referral processes from primary care [163]. Some of the key validation studies supporting these findings are described below:

- SYMPLIFY: One of the validation studies, SYMPLIFY, showed that the MCED test achieved a high specificity of 98.4% and overall sensitivity of 66.3%. Sensitivity was highest for upper gastrointestinal cancers (80.4%), with a negative predictive value (99.1%). Additionally, the predicted accuracy for detecting cancer's site of origin in 84.8% of cases [163].
- THUNDER: Recently, Gao Q *et al.,* 2023, conducted the THUNDER study to evaluate enhanced linear-splinter amplification sequencing for early cancer detection and localization. Two MCDBT models were developed using 161,984 CpG sites and tested on cfDNA from 1693 participants. MCDBT-1 showed 69.1% sensitivity, 98.9% specificity, and 83.2% tissue origin accuracy, with potentially reducing late-stage cancer incidence by up to 46.4% and increasing 5-year survival by up to 40.4%. In contrast, MCDBT-2 had higher sensitivity (75.1%) but lower specificity (95.1%), making it more suitable for higher-risk populations [164]. Additionally, Bryce AH *et al.,* 2023 evaluated a targeted methylation assay using the MCED test for improved cancer detection, reporting high specificity (99.5%), moderate sensitivity (64.3%), CSO prediction (90.3%) and overall sensitivity (84.1%) for gastrointestinal cancer [161].
- PATHFINDER: The PATHFINDER study evaluated the clinical implementation of the CancerSEEK MCED blood test, showing an impressive prediction accuracy of 97% for both initial and subsequent cancers. Nearly half of the non-recurrent cancers were diagnosed at an early stage, with over 70% were cancers included in the standard screening guidelines. In fact, most true positive outcomes received diagnostic resolution within a few months [165]. Furthermore, Vittone, J*. et al.,* 2024 reported that the Galleri MCED test successfully identified early-stage solid organ cancers in three clinical cases, demonstrating its potential to detect early-stage cancers, detect malignancies beyond the scope of USPSTF guidelines and render diagnostic evaluations based on CSO predictions [166].

## Future directions, limitation and concluding remarks

Despite the groundbreaking advancement in integration AI with DNA methylation analysis, the field remains nascent, and requires further technological innovations and clinical validation to maximize its potential. Below, we highlight critical areas for future research and development, focussing on early-stage sensitivity, multi-omics integration, explainable AI, population-specific validation, and ethical considerations.

### Enhancing early-stage sensitivity and specificity

Improving sensitivity for early-stage cancers, which often exhibit low tumor fractions in circulating tumor DNA (ctDNA), remains a key challenge in MCED. Current tests, such as Galleri, achieve only 16.8% sensitivity for stage I cancers, highlighting the need for more robust biomarkers [117]. Future research should focus on identifying methylation patterns unique to early tumorigenesis, such as those associated with pre-malignant lesions or field cancerization could enhance sensitivity. Single-cell methylation profiling (e.g., scBS-seq) could help identify clonal epigenetic alterations before clinical symptoms appearance, enabling earlier tumors detection [74]. Additionally, integrating fragmentomics, such as cfDNA fragment length, end motifs, and nucleosome positioning alongside methylation data could further improve sensitivity, as demonstrated by DELFI’s 73% sensitivity for stage I-II cancers [167].

### Multi-omics integration for comprehensive profiling

Methylation does not operate in isolation; its interplay with genetic mutations, histone modifications, and immune microenvironment changes, all of which play role in tumor evolution. Hybrid models combining methylation with somatic mutations (e.g., *KRAS*, *TP53*), proteomic biomarkers (e.g., *CA-125*, *CEA*), or transcriptomic signatures could enhance diagnostic, classification accuracy and provide a more comprehensive view of tumor biology. For example, Freenome’s MCED test integrates methylation, fragmentomics, and proteomics, achieving 79.2% sensitivity for colorectal cancer [142]. Similarly, spatial multi-omics platforms (e.g., Visium HD) may uncover spatially resolved epigenetic-immune interactions, refining TOO prediction and identifying novel therapeutic targets [168].

### Explainable AI (XAI) frameworks for clinical adoption

The "black-box" nature of AI models remains a major barrier to clinical adoption. Clinicians and regulators require transparent, interpretable frameworks to trust and validate these technologies. Tools like EMethylNET, which links methylation features to gene pathways, and SHAP (SHapley Additive exPlanations), quantifies feature importance, are essential for building trust and understanding model predictions [104]. Regulatory agencies, such as the FDA, are increasingly prioritizing algorithm interpretability in their guidelines, underscoring the need for explainable AI in clinical applications [169]. Based on existing studies, Logistic Regression (LR) and Random Forest (RF) have shown strong potential in MCED tests, with LR offering clinical interpretability and RF handling complex, non-linear data [170]. Future research should focus on developing ensemble models combining algorithm like LR, RF, and other statistical approaches to enhance MCED accuracy and reliability.

### Population-specific validation and global equity

Most MCED tests are validated in Western cohorts, limiting their applicability to diverse populations. For instance, *SEPT9* methylation shows variable performance in Asian vs. European colorectal cancer cohorts, highlighting the need for geographically tailored biomarkers [171]. Moreover, ensuring equity in MCED tests is crucial as they develop, requiring proactive efforts to prevent disparities in access and benefits [14]. Large-scale studies like the Singapore Multi-Cancer Screening Trial (NCT05808300) and SPOT-MAS’s validation in over 10,000 Vietnamese patients [140], demonstrate the importance of population-specific validation. Ensuring global access to these technologies is equally critical. For example, Galleri’s $949 price tag limits its use in low-income countries, necessitating cost-effective alternatives like IMPRESS, which reduces sequencing costs by 70% [46].

### Technological advancements for scalability and precision

A) Single-Cell and Long-Read Sequencing: Technologies such as Single-cell bisulfite sequencing (scBS-seq) and nanopore sequencing could resolve methylation heterogeneity and detect rare tumor clones in ctDNA, improving early detection precision [172].

b) Liquid Biopsy 2.0: In addition to cfDNA, analyzing methylation in extracellular vesicles (EVs) or circulating tumor cells (CTCs) may improve specificity, as shown by Epic Sciences’ CTC-based assay [173].

c) Point-of-Care Testing: Developing portable, low-cost methylation profiling devices could expand access to MCED, particularly in resource-limited settings.

### Ethical, regulatory, and implementation challenges

The rise of AI-driven MCED tests necessitates robust frameworks for data privacy, algorithmic bias mitigation, and equitable access. Regulatory agencies must standardize validation protocols, as current MCED trials (e.g., PATHFINDER, SYMPLIFY) vary in design and endpoints [163, 165]. Additionally, the potential for overdiagnosis and overtreatment, particularly for indolent cancers detected by MCED tests, must be carefully managed.

## Limitations

This review provides an overview of recent advancements in MCED tests but has several limitations. Detailed descriptions of clinical trial designs and validation cohorts were not included, limiting insights into real-world specificity and performance [174, 175]. Key emerging areas, such as epigenetic biomarkers—specifically histone and chromatin modifications—were also not addressed, despite their potential relevance to MCED [176]. Additionally, we did not cover AI-based models utilizing serum protein biomarkers, where real-world data are critical for clinical reliability and generalizability [177]. Multi-omics-based MCED platforms, such as SeekInCare, which show promise in detecting a wide range of cancers, were mentioned but not explored in detail [178]. Ongoing challenges in MCED technologies, such as limited sensitivity for early-stage cancers, undefined follow-up protocols, uncertain insurance coverage, and the need for long-term clinical trials, underscore the need for continued innovation and comprehensive evaluation. These gaps not only highlight the scope and limitations of the review but also point to important directions for future research and clinical development.

## Conclusions

The integration of AI and cancer epigenomics has significantly advanced precision oncology, with DNA methylation biomarkers becoming a key element in non-invasive cancer detection and monitoring methods. Multi-cancer early detection (MCED) tests, such as Galleri and CancerSEEK, demonstrate the clinical potential of methylation biomarkers, offering a revolutionary shift from reactive to proactive cancer management. These technologies have the potential to reduce cancer mortality rates by 30–50%, especially for cancers that currently lack standardized screening procedures [179].

Despite advancements in AI-driven multi-omics integration and epigenetic biomarkers for early cancer detection, challenges remain in improving detection sensitivity, ensuring model interpretability, mitigating demographic biases, and addressing data imbalance. Moreover, the advancement of cancer treatment will rely on joint efforts to integrate multi-omics data, enhance artificial intelligence tool accessibility, and confirm technology effectiveness across global populations. By addressing these aforementioned issues, the next generation of MCED tests has the potential to transform oncology, facilitating early cancer detection, precise treatments, and improved patient outcomes on a global scale.

## Data Availability

No datasets were generated or analysed during the current study.

## Abbreviations

DNMTs: DNA methyltransferases
TET: ten-eleven translocation enzymes
TOO: tumor tissue-of-origin
EMT: Epithelial-to-Mesenchymal Transition
HCC: hepatocellular carcinoma
EMX1-FL: the full-length protein isoform of EMX1
EGFR-ERK: Epidermal Growth Factor (EGF) Receptor-dependent Extracellular-signal Regulated Kinase (ERK)
ALNM: Axillary Lymph Node Metastasis
ER+: Estrogen Receptor Positive
HER2−: Human Epidermal Growth Factor Receptor 2 Negative
CNV: copy number variation
LUAD: lung adenocarcinoma
MeDIP: Methylated DNA immunoprecipitation
UHPLC-MS/MS: ultra-high-performance liquid chromatography combined with mass spectrometry
RRBS: Reduced Representation Bisulfite Sequencing
MSRE: Methylation-Sensitive Restriction Enzyme
EWAS: epigenome-wide association studies
NGS: Next generation sequencing
MRE-seq: methylation-sensitive restriction enzyme sequencing
FFPE: Formalin-Fixed, Paraffin-Embedded
smMIPs: single molecule Molecular Inversion Probes
SVM: Support Vector Machine
GBM: Gradient Boosting Machines
LASSO: Least Absolute Shrinkage and Selection Operator
LR: Logistic Regression
MLR: Multinomial Logistic regression
RF: Random Forests
GLM: Generalized Linear Model
kNN: k-Nearest Neighbors
GCNN: Graph Convolutional Neural Network
WGS: whole-genome sequencing
DMRs: Differentially methylated regions
ACE: alternating current electrokinetics
EV: Extracellular vesicles
MCB: methylation-correlated blocks
HM score: Hypermethylation score
TOTEM: cTdna Origin Tracker dependent on Epigenetic Methylation markers
MFCUP: Methylation-based classifier
CNNs: Convolutional Neural Networks
VAE: Variational autoencoder
GCNNs: Graph Convolutional Neural Networks
NLP: Natural language processing (NLP)
