# Correction: Artificial intelligence in cancer epigenomics: a review on advances in pan-cancer detection and precision medicine

**DOI:** 10.1186/s13072-025-00604-7

**Published:** 2025-07-11

**Authors:** Karishma Sahoo, Prakash Lingasamy, Masuma Khatun, Sajitha Lulu Sudhakaran, Andres Salumets, Vino Sundararajan, Vijayachitra Modhukur

**Affiliations:** 1 Integrative Multiomics Lab, School of Bio Sciences and Technology, Vellore Institute of Technology, Vellore, 632014 Tamil Nadu India; 2 Department of Obstetrics and Gynecology, Institute of Clinical Medicine, University of Tartu, L. Puusepa 8, Tartu, 50406 Estonia; 3 Celvia CC AS, Tartu, 50411 Estonia; 4 Department of Obstetrics and Gynecology, University of Helsinki and Helsinki University Hospital, Haartmaninkatu 8, Helsinki, 00290 Finland; 5 Division of Obstetrics and Gynecology, Department of Clinical Science, Intervention and Technology (CLINTEC), Karolinska Institute, Karolinska University Hospital, Huddinge, 14183 Sweden

**Epigenetics & Chromatin (2025) 18:35**

10.1186/s13072-025-00595-5

In Table 4 of this article, the title was inadvertently omitted.The correct title of Table 4 should be:

“Summary of DNA methylation-based MCED tests: key features and performance metrics from literature and industry sources”.

**Table 4:** Summary of DNA methylation-based MCED tests: key features and performance metrics from literature and industry sources

Company/ Method	Technology	Cancer types Detected	Sensitivity/Specificity/TOO accuracy	Sample Type	AI algorithm	Year/Country	Features	Refs.
IvyGene	Whole genome Methylation Analysis using PCR & NGS	Liver, breast, colorectal, and lung cancers	84%; 90%; N/A	Plasma (ctDNA)	Utilizing AI, combined with cutting edge technology for analysing ctDNA methylation patterns.	USA 2018–2019.	- Offers a non-invasive solution for early cancer detection. - Measures the status of the methylation levels of cfDNA.	[124]
cfMeDIP-Seq	cfDNA Methylated DNA Immunoprecipitation Sequencing	Pancreatic, colorectal, breast, lung, renal, bladder, AML, glioma	AUROC: 0.980 (AML), 0.918 (PDAC), 0.971 (LUC); High; N/A	Plasma (cfDNA)	Incorporates ML (Random Forest) classifiers on cell-free DNA methylation.	Canada; first major publication in 2019– 2020	- Enriches for methylated DNA without bisulfite conversion. - Demonstrates high sensitivity to low fractions of cancer DNA. - Effective for subtype-specific and early-stage cancer detection.	[125, 126]
PanSEER	ctDNA-Methylation Biomarkers & PCR Sequencing	Colorectal, esophageal, liver, lung, and stomach cancers	88.2% (post-diagnosis);95%; N/A	Plasma (cfDNA)	Utilizes a ML algorithm (logistic regression classifier) trained on methylation markers for early detection and TOO prediction.	China/USA collaboration 2020	- Analyzes 477 cancer-specific DMRs with 10,613 CpGs, - Offers high sensitivity down to 0.01% cancer DNA. - Cost-effective with low cfDNA input.	[127]
Galleri	Targeted DNA Methylation Sequencing	Over 50 cancer types	51.5% overall; 16.8% (stage I), 40.4% (stage II), 77.0% (stage III), 90.1% (stage IV); 99.1%; 88.7%.	Plasma (cfDNA)	Advanced ML on targeted methylation data to detect and localize TOO of multiple cancers from cfDNA.	USA; 2018.	- High specificity for low tumor fraction. - Variable sensitivity by cancer type and stage. - Validated in CCGA and PATHFINDER trials.	[128]
CancerSEEK/Exact Science (USA)	Mutation and Protein Biomarker Analysis; Liquid Biopsy using cfDNA and ML for cancer prediction	About 8 cancer types (ovary, liver, stomach)	62% (1005 as number of patient with cancer); 99.1%; 63%.	Plasma (ctDNA)	ML classifier combining ctDNA mutations and selected protein biomarkers.	USA; 2019–2020.	- Integrates multi-omics. - Detects cancers without standard screening.	[123, 129]
Adela Bio/Adela	Cell-free methylated DNA immunoprecipitation-sequencing.	About 7 cancer types	70%–75%; 99%; 91%.	Plasma (cfDNA)	ML application analyzing methylome signals	USA; 2021	- Avoids bisulfite conversion; - Effective for early-stage and subtype-specific detection; - Utilizes ML algorithms to identify key methylated regions in cfDNA.	[130]
TR(ACE) /Biological dynamics	Alternating current electrokinetics (ACE) platform to purify extracellular vesicles from plasma; ML algorithm.	About 7 cancer types	Sensitivity of 71.2% (95% CI: 63.2–78.1); 99.5% ; 43.8% - 95.5%	Circulating extracellular vehicles (EVs)	Uses ML classifier on extracellular vesicles (EV)–associated biomarkers.	USA; 2020–2021	- Utilizes ACE platform to isolate circulating EVs from plasma and is used for multi-marker analysis. - Efficient analysis of a large number of samples.	[131]
Cancer detector	cfDNA bisulfite sequencing, probabilistic model.	Study report on liver cancer but claims to detect all types of cancers.	94.8%; 100%; N/A.	Plasma (cfDNA)	NA	USA; 2018	- Developed CancerDetector which focuses on joint methylation states improves sensitivity for detecting abnormal cfDNAs. - Achieves high accuracy and its prediction is consistent with clinical information.	[132]
EpiPanGI Dx	Bisulfite sequencing method and Machine learning	GI cancers (CRC, pancreatic, stomach)	AUC: 0.88; 96%; 0.85–0.95.	Plasma (cfDNA)	Methylation-based biomarker approach with machine learning for GI cancer detection.	U.S., Germany, Japan, South Africa, and Spain; 2020–2021.	- This test identified the three distinct DMR panels that are Cancer-Specific Biomarker Panels. - Pan-GI Cancer Panel and multi-cancer TOO prediction panel.	[133]
Burning Rock Dx	Targeted methylation sequencing assay combined with machine learning.	6 Cancers (liver, colon/rectum, esophagus, pancreas, lung and ovary)	80.6%; 98.3%; 81.0%.	Plasma (cfDNA)	- ML approaches (SVM) for ctDNA methylation or targeted gene panel detection. - Multi-class logistic regression was used to predict tissue origin.	China; 2020	- Optimized for low-depth sequencing; validated in THUNDER-II trial. - Shows high specificity and accurate TOO prediction.	[134]
GENECAST	Targeted methylation sequencing	14 cancer types	72.86% ; 96.67% (AUC = 0.86); N/A.	Plasma (cfDNA)	N/A	China; 2019–2021	- The model developed was based on 37 MCB. Biomarker methylation differences were computed using a HM-score.	[135]
Guardant Reveal	Methylation panel (500 CpGs) + fragmentomics + ML	13 cancers	76.4%/42%; 97.9%; 82%.	ctDNA	Statistical and ML–based algorithm for ctDNA signals (both genomic, epigenomic and fragmentomics).	Usa; 2021	- Combines methylation and fragment size analysis. - FDA-approved for colorectal cancer recurrence.	[136]
DELFI (Delfi DX)	Genome-wide cfDNA fragmentation + ML	7 cancers (lung, breast, liver)	73% (Stage I–II); 98%; 85%.	Plasma (cfDNA)	ML on cfDNA fragmentomics (fragmentation profiles).	USA; 2019	- Low-cost WGS approach. - Detects fragmentation patterns linked to chromatin instability.	[137]
OncoSeek	Protein biomarkers (CA-125, CEA) + AI	9 cancers	51.7%; 95%; 66.8%.	Plasma (serum)	Uses AI for calculating the POC index	California, USA;	- Low-cost protein-based test. - Integrates clinical metadata for risk stratification.	[138]
SPOT-MAS (Gene Solutions)	Targeted methylation (14 genes) + ML	5 cancers (breast, liver, CRC, lung)	78%; 99.8%; 84%	ctDNA	Integrates ML analysis on ctDNA mutation and methylation signals.	Vietnam; 2021–2022	- Validated in 10,000+ Vietnamese patients. - Optimized for low-resource settings.	[139, 140]
SeekInCare (SeekIn)	Methylation + CNVs + ML	20 cancers (NSCLC, CRC, liver)	65.5%; 97%; 93%	Plasma (cfDNA)	Deals with Multi-omic (ctDNA + protein) data; gradient-boosting machine ML algorithms for early detection and surveillance.	China; 2020–2021.	- Resource-optimized cancer screening for large populations.	[141]
Freenome	Methylation + fragmentomics + proteomics + gradient boosting	8 cancers (CRC, lung, breast)	79.2%; 92% in CRC; N/A.	Plasma (cfDNA)	ML enabled multi-omics	USA; 2019	- Multi-omics approach; - This is under clinical evaluation in PROSPECT study (NCT05581476).	[142]

*a. ACE: alternating current electrokinetics, b. EV: Extracellular vesicles, c. MCB: methylation-correlated blocks, d. HM score: Hypermethylation score; e. WGS: Whole genome sequencing. f. SVM: Support Vector Machine., g. POC: probability of cancer; h. ML: Machine learning, i. AI: Artificial Intelligence.; f. N/A- Not available

The original article has been corrected.

